# Potent Phototoxicity of Marine Bunker Oil to Translucent Herring Embryos after Prolonged Weathering

**DOI:** 10.1371/journal.pone.0030116

**Published:** 2012-02-01

**Authors:** John P. Incardona, Carol A. Vines, Tiffany L. Linbo, Mark S. Myers, Catherine A. Sloan, Bernadita F. Anulacion, Daryle Boyd, Tracy K. Collier, Steven Morgan, Gary N. Cherr, Nathaniel L. Scholz

**Affiliations:** 1 Environmental Conservation Division, National Oceanic and Atmospheric Administration, Northwest Fisheries Science Center, Seattle, Washington, United States of America; 2 University of California-Davis Bodega Marine Laboratory, Bodega Bay, California, United States of America; 3 Departments of Environmental Toxicology and Nutrition, University of California Davis, Davis, California, United States of America; Institute of Marine Research, Norway

## Abstract

Pacific herring embryos (*Clupea pallasi*) spawned three months following the Cosco Busan bunker oil spill in San Francisco Bay showed high rates of late embryonic mortality in the intertidal zone at oiled sites. Dead embryos developed to the hatching stage (e.g. fully pigmented eyes) before suffering extensive tissue deterioration. In contrast, embryos incubated subtidally at oiled sites showed evidence of sublethal oil exposure (petroleum-induced cardiac toxicity) with very low rates of mortality. These field findings suggested an enhancement of oil toxicity through an interaction between oil and another environmental stressor in the intertidal zone, such as higher levels of sunlight-derived ultraviolet (UV) radiation. We tested this hypothesis by exposing herring embryos to both trace levels of weathered Cosco Busan bunker oil and sunlight, with and without protection from UV radiation. Cosco Busan oil and UV co-exposure were both necessary and sufficient to induce an acutely lethal necrotic syndrome in hatching stage embryos that closely mimicked the condition of dead embryos sampled from oiled sites. Tissue levels of known phototoxic polycyclic aromatic compounds were too low to explain the observed degree of phototoxicity, indicating the presence of other unidentified or unmeasured phototoxic compounds derived from bunker oil. These findings provide a parsimonious explanation for the unexpectedly high losses of intertidal herring spawn following the Cosco Busan spill. The chemical composition and associated toxicity of bunker oils should be more thoroughly evaluated to better understand and anticipate the ecological impacts of vessel-derived spills associated with an expanding global transportation network.

## Introduction

Pacific herring (*Clupea pallasi*) and other shore-spawning forage fish are commercially and ecologically important species in coastal marine ecosystems. Because their embryos incubate attached to substrates in shallow nearshore areas, they are highly vulnerable to the harmful effects of oil spills that lead to shoreline contamination. Herring embryos spawned three months following the Cosco Busan oil spill in San Francisco Bay showed high rates of late embryonic mortality in the intertidal zone at oiled sites [1]. Although affected embryos developed to the hatching stage (e.g. fully pigmented eyes) by the time of sampling, microscopic examination revealed that most embryos were in a state of deterioration, with severe tissue disintegration. In contrast, embryos incubated beneath at least 1 m of turbid water in the subtidal zone at oiled sites showed evidence of only sublethal oil exposure (cardiotoxicity associated with polycyclic aromatic compounds, PACs [2], [3], [4], [5]). Therefore, distinct mechanisms of oil toxicity occurred at different water depths, and the enhanced toxicity in the intertidal zone may have been induced through an interaction between oil and another environmental stressor. Such an interaction between sunlight and PACs (or other oil compounds) could change toxicity by either of two known mechanisms. Some PACs produce phototoxic reactions in cells and tissues, through activation by ultraviolet (UV) radiation and generation of reactive oxygen species that cause membrane damage [6], [7]. In laboratory studies with diverse aquatic animals, this UV-enhanced phototoxicity of PACs (i.e. photosensitization) occurs at much lower tissue concentrations than produce acute toxicity in the absence of UV light [8]. A second potential mechanism is through photomodification of PACs by UV-mediated oxidation before they are taken up and accumulated by the organism. Some quinone photooxidation products of PACs increase toxicity to aquatic invertebrates relative to the parent compounds [9].

**Figure 1:**
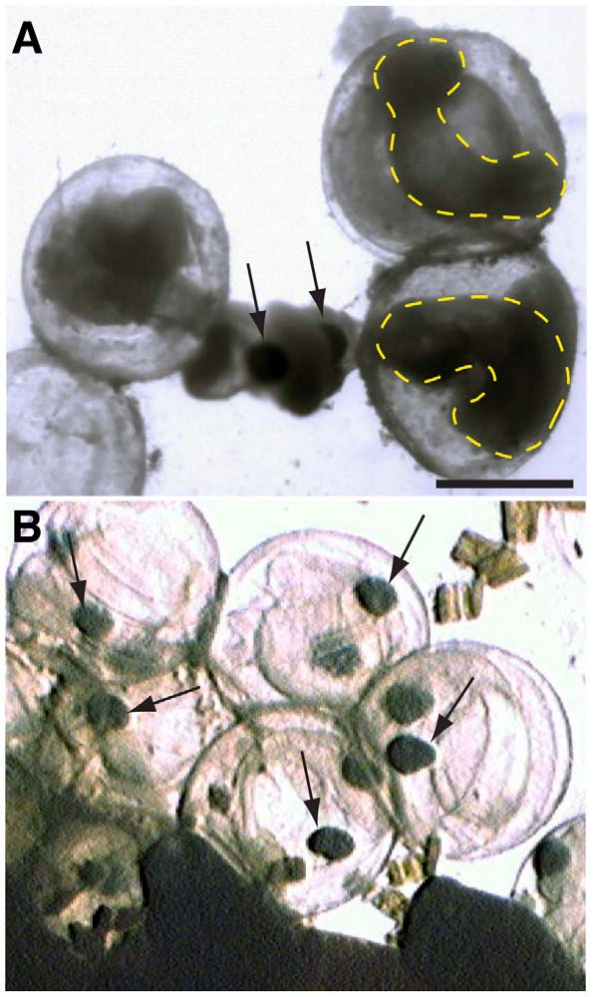
Morphological condition of Pacific herring embryos collected from field sites in San Francisco Bay in February 2008. Sampling and imaging of embryos is described elsewhere (1). (A) Opaque dead embryos within chorions from a site (Sausalito waterfront) affected by the Cosco Busan spill. Arrows indicate pigmented eyes on an embryo protruding from a ruptured chorion, while yellow dashed lines outline the “ghosts” of necrotic late embryos. (B) Embryos within chorions from the same site after recovery in February 2010. Viable hatching-stage embryos are translucent, with pigmented eyes indicated by arrows. Scale bar 1 mm.

While exposure of fish embryos to relatively low concentrations of oil generally results in sublethal cardiotoxicity (from non-phototoxic PACs), a phototoxic interaction with sunlight is one mechanism that can lead to acute and overt mortality. Previous studies showed that exposure to unrefined crude oil and sunlight or UV light alone produced lethal photosensitization in Pacific herring larvae at tissue PAC concentrations that were sublethal (i.e. cardiotoxic) in the absence of UV [10]. Toxicity required exposure to crude oil before sunlight and was associated with uptake of unmodified PACs, indicating that a phototoxic reaction occurred within tissues rather than a photomodification mechanism. However, the most highly phototoxic, conventionally measured PACs (e.g. anthracene, fluoranthene, pyrene) were present at very low concentrations, suggesting the presence of other photosensitizing compounds. Bunker oils that power large vessels such as the Cosco Busan contain the highly concentrated residuum of the crude oil refining process, and are thus more chemically complex than unrefined crude oil. Using zebrafish embryos, we recently showed that bunker oils have an even higher phototoxic potential than unrefined crude oil. Sequential exposure to relatively high concentrations of unweathered bunker oils and sunlight caused mortality that was not observed with an equivalent concentration of crude oil [11]. However, as for unrefined crude oil [10], photomodification was not required for this activity, and although the bunker oils had higher concentrations of known phototoxic PACS, phototoxicity could not be attributed to the conventionally measured suite of PACs [11]. The morphological and cellular effects leading to lethality from crude oil phototoxicity in fish embryos or larvae are not well understood. The extensive necrotic damage apparent in herring embryos collected from oiled site intertidal zones following the Cosco Busan spill is most consistent with a cytolytic process such as plasma membrane damage following UV-enhanced phototoxicity. The phototoxicity of unweathered bunker oils was manifested in zebrafish as an acutely lethal cellular necrosis, leading to the entire disintegration of embryos in severe cases [11]. Nevertheless, this interaction between sunlight and residual oils has not been demonstrated to occur in non-model fish species after prolonged weathering at low, environmentally relevant concentrations. This includes the population of Pacific herring that spawned at oiled intertidal locations in San Francisco Bay following the 2007 Cosco Busan bunker spill. Establishing the cause of this toxicity is particularly important because the high degree of embryo lethality following the spill was unexpected, and the spill occurred in a complex urban environment with multiple sources of potentially toxic contaminants. Here, we tested whether combined exposure to sunlight and trace levels of weathered Cosco Busan bunker oil was sufficient to produce the lethal tissue damage in herring embryos that was observed three months following the Cosco Busan spill (Fig. 1), and in photosensitization reactions in zebrafish embryos at higher concentrations of unweathered bunker oil [11].

## Results and Discussion

### Overview of experimental design

We used oiled gravel columns to generate a contaminated effluent that mimics the natural weathering of oil by water percolating through shoreline substrates with tidal exchange [3], [12], [13], [14], [15]. Herring embryos were produced from gametes obtained by capturing ripe adults from the San Francisco Bay spawning population. We weathered the oiled gravel with continuously flowing seawater (Fig. S1A, B) beginning 14 January 2009, and exposed herring embryos in sequential experiments starting on 22 January, 26 February, and 18 March. Oil weathering and embryo exposures were conducted outdoors (Fig. S1C) to assess the influence of sunlight on toxicity, and we compared equivalent mass loadings of Cosco Busan bunker oil (CBBO) to a reference crude oil (Alaska North Slope crude oil; ANSCO) at 0.1, 0.3 and 1.0 g oil per kg gravel. Columns (including negative controls) were randomly distributed among six water tables and covered by either UV-transmitting or UV-reducing plastic hoods such that triplicates of each dose were assigned to either full-spectrum sunlight or reduced UV sunlight (Fig. S1A). For the 26 February and 18 March exposures, we measured PACs in water samples collected at the beginning and end of incubation and in embryos at the end of each exposure interval (8 days post-fertilization, dpf).

### ANSCO and CBBO both produce canonical PAC-mediated cardiotoxicity in embryos protected from UV co-exposure

**Figure 2:**
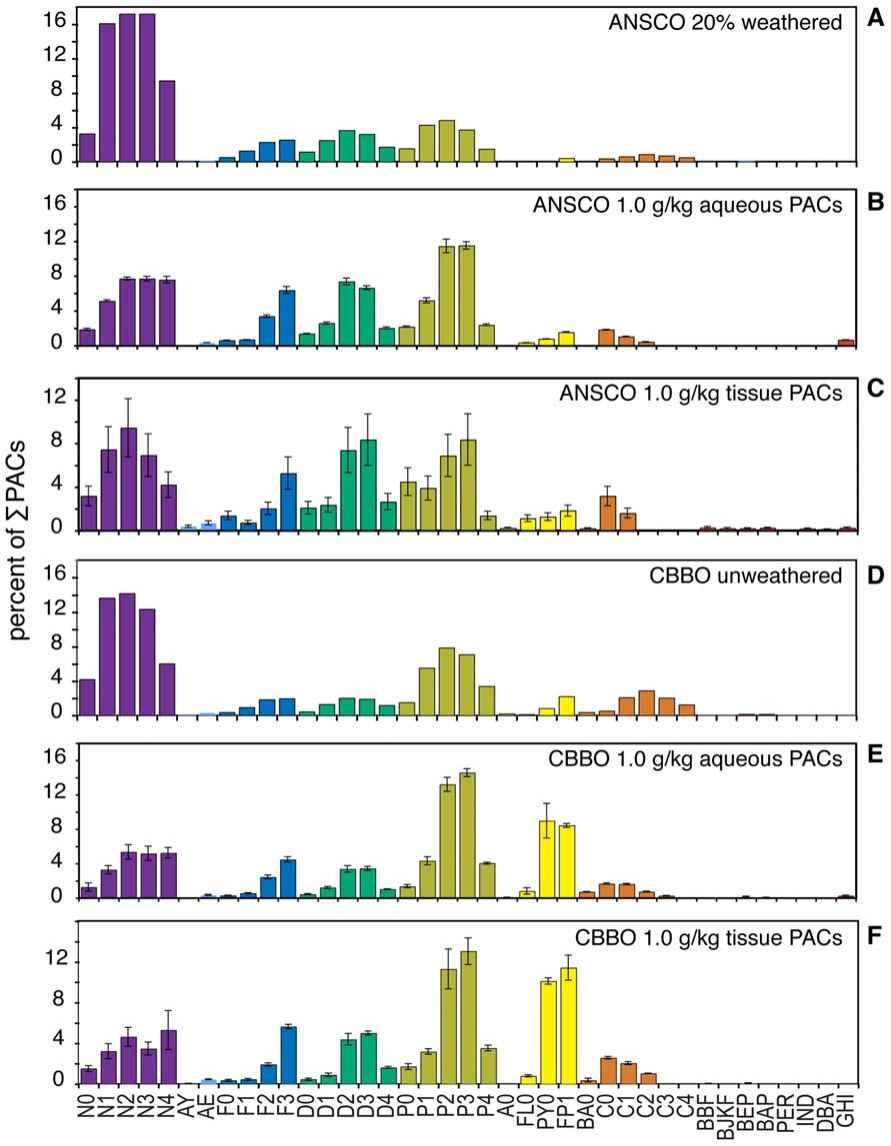
PAC composition of oils, oiled gravel effluent, and exposed herring embryos. Composition of PACs determined by GC/MS represented as percent of summed PACs for each of 39 analytes. Data for oiled gravel effluent and embryos are shown only for the highest dose of oil under UV blocking conditions from the 26 February experiment, but are representative of the general patterns observed in all treatments. Data from whole oils are derived from a single determination. Data for effluent and embryos are the mean and s.e.m. of three determinations. (A) Whole 20% weathered ANSCO that was applied to gravel. (B) Aqueous PACs in effluent from 1.0 g/kg ANSCO gravel. (C) Tissue PACs in embryos exposed to effluent in B. (D) Whole unweathered CBBO applied to gravel. (E) Aqueous PACs in effluent from 1.0 g/kg CBBO gravel. (F) Tissue PACs in embryos exposed to effluent in E. N, naphthalenes; AY, acenaphthylene; AE, acenaphthene; F, fluorene; D, dibenzothiophene; P, phenanthrene; A, anthracene; FL, fluoranthene; PY, pyrene; FP, fluoranthenes/pyrenes; BA, benz[*a*]anthracene; C, chrysene; BBF, benzo[*b*]fluoranthene; BJKF, benzo[*j*]fluoranthene/benzo[*k*]fluoranthene; BEP, benzo[e]pyrene; BAP, benzo[a]pyrene; PER, perylene; IND, indeno[1,2,3-*cd*]pyrene, DBA, dibenz[*a*,*h*]anthracene/dibenz[*a*,*c*]anthracene; GHI, benzo[*ghi*]perylene. Parent compound is indicated by a 0 (e.g., N0), while numbers of additional carbons (e.g. methyl groups) for alkylated homologs are indicated as N1, N2, etc.

Water concentrations of ∑PACs were very low (highest dose 1800 ng/L or 1.8 parts per billion) at the beginning of embryo incubation for each experiment (Table 1, Fig. S2) For the 26 February and 18 March exposures, respectively, background levels of PACs in the negative control effluents for the UV-reducing treatment groups had upper 95% confidence limits of 87 and 82 ng/L for aqueous summed PACs (∑PACs), and 16 and 23 ng/L sum parent and alkylated tricyclic PACs (∑TACs; fluorenes, dibenzothiophenes, phenanthrenes). Effluents from all oiled gravel doses had aqueous ∑PACs and ∑TACs that were well above the upper 95% confidence limits of the controls. The composition of PACs in oiled gravel effluent showed a weathered pattern for both oils (Fig. 2). As weathering progressed, parent and lower molecular weight alkylated PACs became relatively depleted. For ANSCO, this led to an overall depletion of the naphthalene series and a proportional increase in the C2- and C3-alkylated fluorene, dibenzothiophene, and phenanthrene homologues (Fig. 2B). This was evident, for example, in a shift of the ratio of summed alkyl-naphthalenes to summed alkyl-phenanthrenes from 1.3 in whole oil to 0.9 in aqueous PACs. The CBBO weathering pattern was similar (Fig. 2E), but with much higher relative proportions of pyrene and alkyl-fluoranthenes/pyrenes and a larger shift in the ratio of alkyl-naphthalenes to alkyl-phenanthrenes (1.9 to 0.5). For both oils, PAC uptake by embryos closely mirrored the PAC composition in the column effluents (Fig. 2C and F, ANSCO and CBBO, respectively). The patterns for water and tissues also were similar for both oils at the lower doses (Fig. S3), with more weathering generally evident at successive exposure intervals (Fig. S2). Cleaning algae from the gravel between the 26 February and 18 March experiments most likely produced an exception to the expected pattern (higher levels of naphthalenes) by exposing unweathered gravel surfaces (Fig. S2B).

**Table 1:** Aqueous PAC levels in column effluents under UV-reducing plastic.

Experiment	Dose	∑PACs^1^	∑TACs
26 February exposure			
	clean	69±18	11±2
	urban	56±9	14±2
	control upper 95% CL	87	16
	ANSCO 0.1	130±25	62±6
	ANSCO 0.3	230±10	125±3
	ANSCO 1.0	507±52	322±41
	CBBO 0.1	270±20	149±13
	CBBO 0.3	620±31	368±19
	CBBO 1.0	1467±120	797±48
18 March exposure			
	clean	62±5	11±3
	clean upper 95% CL	82	23
	CBBO 0.1	620±105	334±43
	CBBO 0.3	1100±0	496±7
	CBBO 1.0	1767±67	624±31

1 Values are ng/L, mean ± s.e.m. (except upper 95% confidence limits, CL).

**Figure 3:**
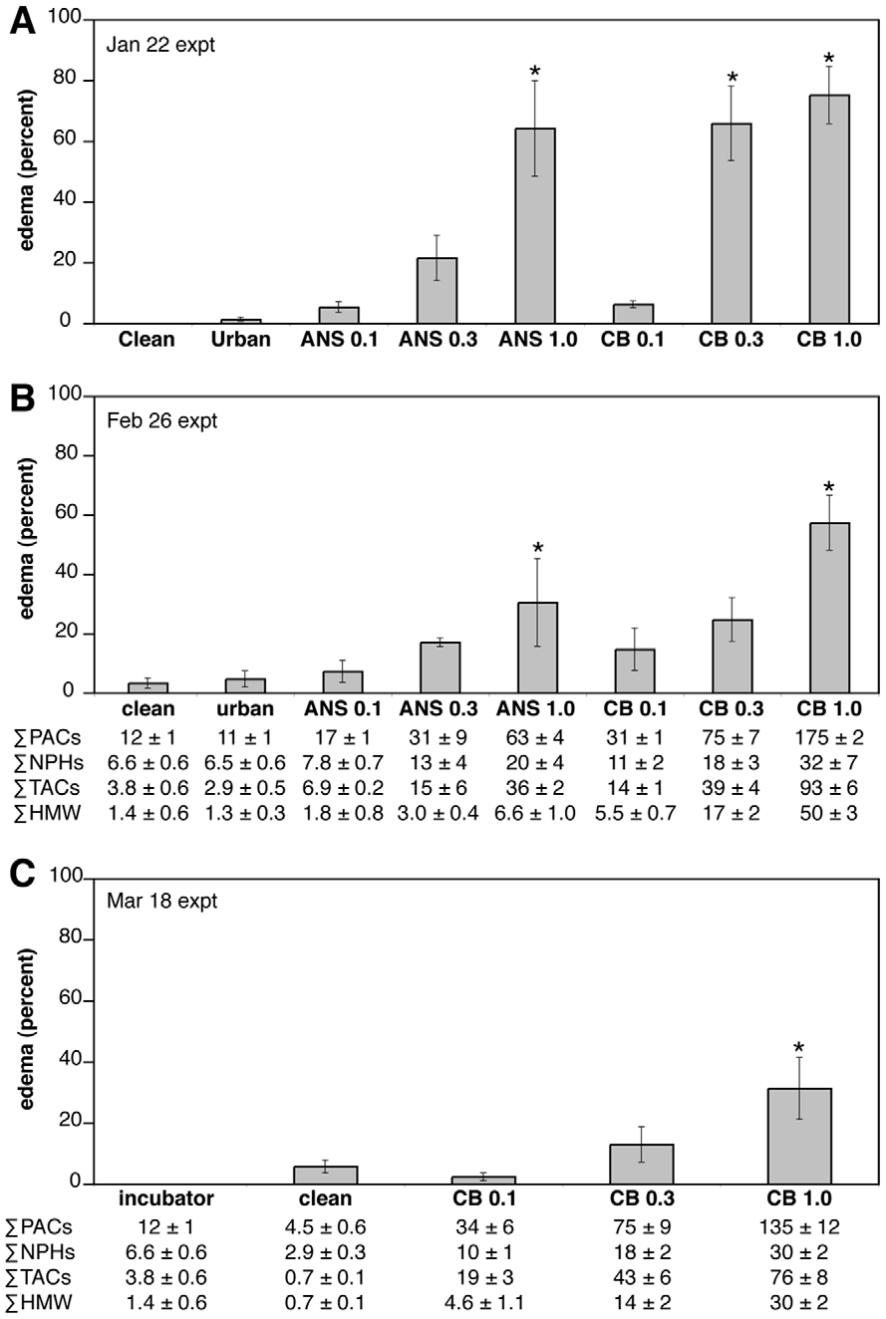
Dose-dependent pericardial edema in herring embryos after oil exposure under UV-reducing plastic. Edema was quantified in live, hatched larvae. Values represent the mean percent ± SEM from three replicates for each control or oil dose for the experiments starting 22 January (A), 26 February (B) and 18 March (C). Nominal oil loadings (0.1, 0.3, and 1.0 g/kg) are indicated for each oil (ANSCO, *ANS*; CBBO, *CB*). Tissue PACs are shown for the 26 February and 18 March experiments as sum total (∑) PACs, sum parent and alkylated naphthalenes (NPHs), sum parent and alkylated tricyclic compounds (TACs; fluorenes, dibenzothiophenes, phenanthrenes), and sum high molecular weight compounds (HMW; fluoranthene, pyrene, C1-fluoranthene/pyrenes, benz[*a*]anthracene, chrysene, benzo[*b*]fluoranthene, benzo[*j*]fluoranthene/benzo[*k*]fluoranthene, benzo[e]pyrene, benzo[a]pyrene, perylene, indeno[1,2,3-*cd*]pyrene, dibenz[*a*,*h*]anthracene/dibenz[*a*,*c*]anthracene, and benzo[*ghi*]perylene).

In the first two experiments, we compared exposure to equivalent oil mass loadings of ANSCO and CBBO. ANSCO served as a positive control to reproduce canonical crude oil cardiotoxicity, as a context for identifying possible novel effects from residual oil in CBBO. As expected from prior studies [3], [12], [14], dose-dependent pericardial edema was observed in hatched larvae following exposure to oiled gravel effluent under UV-reducing conditions (Fig. 3). Incidence of edema generally correlated with declining water and tissue PAC concentrations. In the first experiment starting 22 January, the incidence of larval edema was 64±17% after exposure to ANSCO 1.0 g/kg gravel effluent, and 66±12% and 75±9% in larvae exposed to CBBO 0.3 g/kg and 1.0 g/kg gravel effluent, respectively (Fig. 3A). Over the next month of weathering, aqueous PAC concentrations were expected to decline [15]. Indeed, herring embryos from the exposure beginning 26 February showed a decline in edema to 30±15% for exposure to ANSCO 1.0 g/kg, with an embryonic tissue ∑PAC concentration of 63±4 ng/g (Fig. 3B). Exposure to the equivalent mass loading of CBBO resulted in a higher incidence of edema (57±9%) with a tissue ∑PAC concentration of 175±2 ng/g. The CBBO 0.3 g/kg loading produced toxicity similar to ANSCO at 1.0 g/kg (25±7% edema and ∑PAC 75±7 ng/g). Although remixing of the gravel and higher flow rates in the 18 March experiment resulted in higher ∑PAC concentrations, largely due to an increase in naphthalenes (Fig. S2), tissue ∑TACs and higher molecular weight compounds (∑HMW) at the highest CBBO dose were 82% (76±8 ng/g) and 60% (30±2 ng/g) lower, respectively, and the incidence of edema was significantly lower at 31±10% (Fig. 3C; t test, α = 0.05). The upper 95% confidence limits for the incidence of edema in negative controls was 8% for the 26 February experiment and 15% for the 18 March experiment. Based on the incidence of edema in the UV-reduced, oil-exposed groups in both experiments, we estimate that the EC_20_ for cardiotoxicity in herring embryos is in the range of 300–1000 ng/L ∑PACs or 200–500 ng/L ∑TACs. Although this cardiotoxicity is sublethal in embryos, edema in embryos results in delayed larval mortality due to poor feeding [2].

### CBBO but not ANSCO interacts with full-spectrum sunlight to produce acutely lethal necrosis at very low water and tissue PAC concentrations

**Figure 4:**
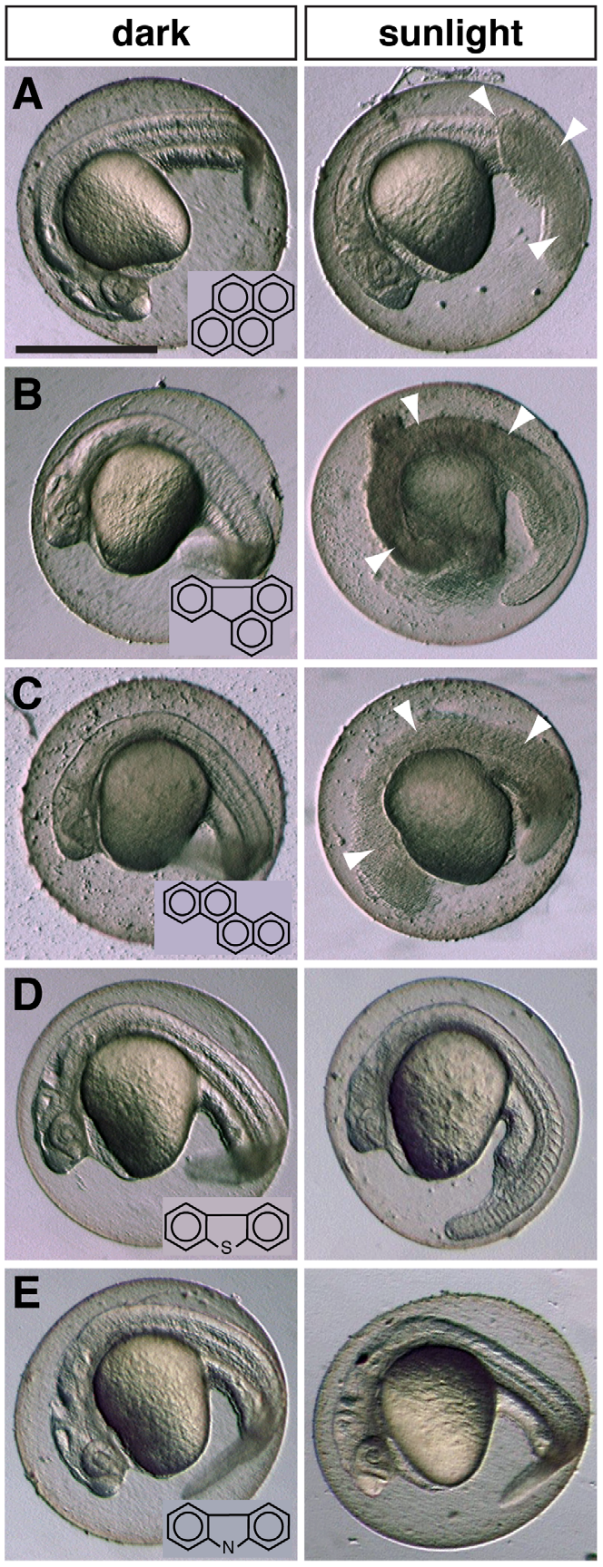
4-ring PACs in CBBO are phototoxic, but 3-ring heterocyclic PACs are not. Zebrafish embryos were exposed from 4–24 hours post-fertilization to 50 µM (A) pyrene, (B) fluoranthene, (C) chrysene, (D) dibenzothiophene and (E) carbazole, followed by 20 min sunlight exposure. Left panels show PAC-exposed maintained in darkness, right panels show subsamples exposed to ambient sunlight. Arrowheads indicate necrotic regions. PAC structures are shown in the insets. Scale bar is 0.5 mm.

Because there are few studies that systematically tested the phototoxic potential of different individual PACs, and none specifically in fish embryos, we determined the potential for a suite of known CBBO PACs to reproduce the type of photosensitization reaction we observed in zebrafish embryos previously with unweathered bunker oils [11]. Tests with cell lines showed that PAC phototoxicity generally requires at least a 4-membered ring system [16], and studies with crude oil have implicated heterocyclic compounds, e.g., PACs with nitrogen, sulfur or oxygen incorporated into the ring system [10]. Our previous analysis showed that bunker oils are particularly enriched with 3-ringed heterocyclic carbazoles [11]. Using an identical approach, zebrafish embryos were exposed to individual PACs (3-ringed dibenzothiophene and carbazole; 4-ringed pyrene, fluoranthene, and chrysene) from shortly after fertilization to 24 hours post-fertilization (about half-way to hatching stage), then transferred into clean water and exposed to sunlight outdoors. Sunlight exposure for 20 minutes resulted in a rapid lysis of tissues in embryos exposed to pyrene, fluoranthene, and chrysene, (Fig. 4A, B, C) but not dibenzothiophene (Fig. 4D) or carbazole (Fig. 4E). Therefore, among the known PACs in CBBO, pyrene, fluoranthene and chrysene (and their alkylated homologs) are the only known compounds present that would be predicted to contribute to phototoxicity.

**Figure 5:**
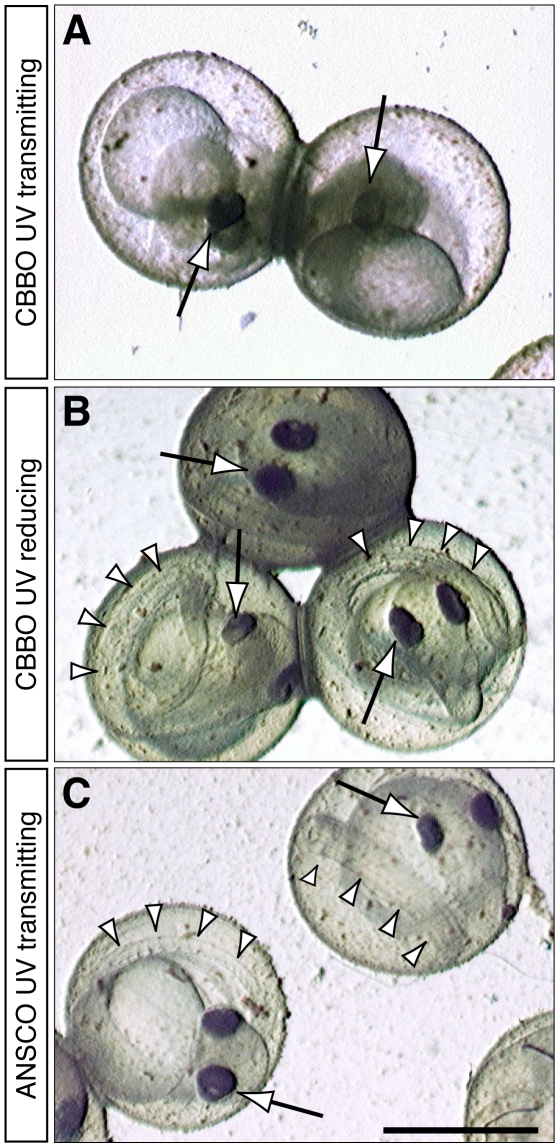
Late embryonic necrosis in herring resulting from CBBO exposure under UV-transmitting plastic is prevented by UV-reducing plastic. Representative embryos from the 22 January experiment are shown at 8 dpf within intact chorions. (A) CBBO 1.0 g/kg dose under UV-transmitting plastic. (B) CBBO 1.0 g/kg dose under UV-reducing plastic. (C) ANSCO 1.0 g/kg dose under UV-transmitting plastic. Arrows indicate pigmented eyes on each embryo, arrowheads in B and C indicate free portion of tail coiled around the yolk sac, which is absent in A. Scale bar is 1 mm.

For logistical reasons (detailed below) we tested CBBO phototoxicity to herring embryos with simultaneous oil and sunlight exposures starting shortly after fertilization. Consistent with our observations in zebrafish embryos [11], co-exposure of herring embryos to CBBO and full-spectrum sunlight resulted in cytotoxicity and the effective disintegration of entire embryos (Fig. 5A). Although not characterized at the cellular level, this response is consistent with phototoxic cellular membrane damage by reactive oxygen species generated from UV-mediated activation of bioaccumulated compounds [17], [18], [19]. This effect was prevented by incubation under UV-reducing plastic (Fig. 5B), and was absent in embryos exposed to ANSCO under UV-transmitting plastic (Fig. 5C). We characterized the time course of CBBO phototoxicity in the 22 January experiment, during the initial weathering of the columns. Exposure to oil and sunlight was initiated the same day as fertilization and by 6 days post-fertilization (dpf) embryos had progressed to the late segmentation stage (50+ somites) with the beginning of eye pigmentation. Embryos exposed to CBBO under UV-transmitting plastic were viable, but showed distinct tailbud defects that were not observed in control (unexposed) or ANSCO-exposed embryos (Fig. S4). Viability continued through 7 dpf and then a loss of epithelial integrity and mortality occurred abruptly by 8 dpf.

**Figure 6:**
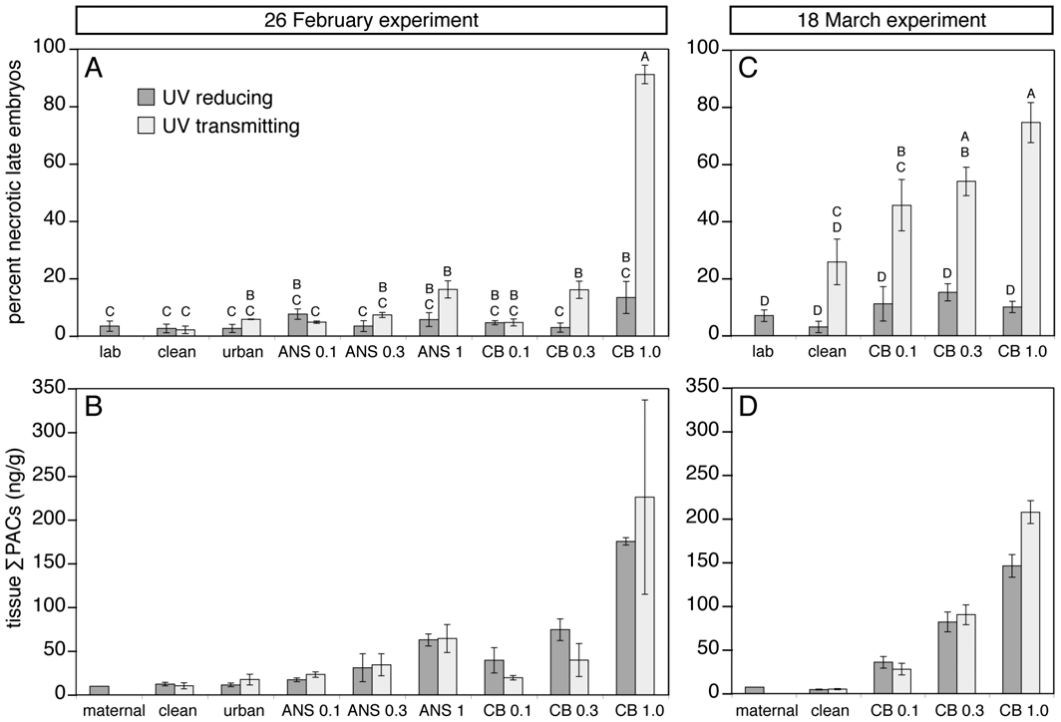
Late embryonic necrosis in herring resulting from CBBO exposure under UV-transmitting plastic is dependent on oil dose. Embryos were exposed to column effluents from fertilization through 8 dpf, and necrotic late embryos and tissue ∑PAC concentrations were quantified. Each graph shows mean ± s.e.m. of three replicates per treatment. (A) Percent necrotic late embryos observed in the 26 February experiment. (B) Corresponding tissue ∑PAC concentrations for the 26 February experiment. (C) Percent necrotic late embryos observed in the 18 March experiment. (D) ∑PAC concentrations measured in corresponding tissue for the 18 March experiment. Letters designate exposure groups that are significantly different from each other.

In the subsequent two experiments, both the incidence of necrotic embryos and tissue concentrations of PACs were quantified at 8 dpf after continuous exposure from fertilization (Fig. 6). In the middle experiment (26 February), the highest CBBO dose (1.0 g/kg gravel) produced 91±3% mortality at 8 dpf in the UV-transmitting exposure, but only 13±6% mortality in the UV-reducing exposure (Fig. 6A). The latter was not significantly different from a 2–6% rate of mortality among embryos incubated in the effluents from clean gravel columns or columns containing unoiled gravel from an urban intertidal location within San Francisco Bay. Both the middle CBBO dose (0.3 g/kg) and highest ANSCO dose (1.0 g/kg) under UV-transmitting conditions produced a small but statistically significant elevation in mortality (each at 16±3%). Tissue concentrations of ∑PACs resulting from the 1.0 g/kg CBBO dose were 225±111 ng/g and 175±4 ng/g for UV-transmitting and UV-reducing treatments (Fig. 6B), respectively, while the estimated UV doses were 0.172 W·hr/cm^2^ and 0.072 W·hr/cm^2^ (Table 2 and Table S1). In the third and final exposure (18 March), the 1.0 g/kg CBBO dose produced 75±7% mortality in the UV-transmitting exposure, but only 10±2% mortality in the UV-reducing exposure (Fig. 6C). Relative to the preceding 26 February experiment, tissue ∑PACs were essentially equivalent (Fig. 6D; 208±31 ng/g for UV-transmitting vs. 146±13 ng/g for UV-reducing). However, the UV dose was almost two times higher (0.321 W·hr/cm^2^ under UV-transmitting plastic, Table 2) due to longer day length and decreased cloud cover. Although embryos incubated in clean gravel effluent showed higher late mortality under UV-transmitting plastic (26±8%), this was not statistically different than the UV-reduced controls (Fig. 6D) and may have been caused by increasing diatom growth coincident with longer day length (see Materials and methods and Fig. S8). In both experiments, a statistically significant increase in late embryonic mortality was observed with exposure to the 0.3 g/kg CBBO dose under UV-transmitting plastic (Fig. 6A and C).

**Table 2:** Estimates of daily UV exposure for 26 February and 18 March experiments.

	Day Length (hr)	Daily UV	
Date		Peak (µW/cm^2^)^1^	Total (µW/cm^2^)^2^
2/26/09	11.26	4240	30399
2/27/09	11.30	3839	27621
2/28/09	11.34	640	4621
3/1/09	11.38	137.5	996
3/2/09	11.42	1875	13633
3/3/09	11.46	2515	18351
3/4/09	11.50	5045	36940
3/5/09	11.54	779.5	5728
3/6/09	11.58	4520	33329
Experiment total			171619
3/18/09	12.07	4157	31944
3/19/09	12.11	4593	35414
3/20/09	12.15	4290	33188
3/21/09	12.19	1053	8174
3/22/09	12.23	5793	45117
3/23/09	12.27	4910	38367
3/24/09	12.32	4607	36120
3/25/09	12.36	5570	43813
3/26/09	12.40	6143	48479
Experiment total			320616

1 measured beneath UV-transmitting plastic covers near peak solar radiation (1–2 PM).

2 calculated with the formula peak UV×day length/π×2.

The tissue levels of phototoxic PACs were calculated as the sum of values for pyrene, fluoranthene, C1-fluoranthene/pyrenes, chrysene, and C1- through C4-chrysenes (Table 3). A dose metric for PAC phototoxicity to fish embryos (medaka, *Oryzias latipes*) was previously established for fluoranthene [20], and factors both UV dose and tissue PAC concentration. Using the range of LD_50_ values estimated by those authors from their medaka data and other published data on the phototoxicity of anthracene and fluoranthene to invertebrate and fish early life history stages, we estimated the LC_50_ range for tissue levels of phototoxic PACs at the estimated UV doses for the February 26 and March 18 exposures (Table 3). We also estimated these values for the previously published studies on ANSCO phototoxicity to Pacific herring embryos and larvae [10]. In each experiment described here, and in the previous study with ANSCO, high levels of lethal phototoxicity (e.g. >80% mortality) were associated with a combination of UV exposure and phototoxic PAC levels that were near or below the estimated LC_50_ value (Table 3).

**Table 3:** Tissue concentrations of phototoxic PACs compared to estimated phototoxic doses.

Experiment	Treatment	∑Phototoxic PACs^1^	Estimated Phototoxic LC_50_	Phototoxicity observed
Feb 26 Experiment			20–260	
	ANSCO 0.3 g/kg UV-r	3.2±0.5		no
	ANSCO 0.3 g/kg UV-t	2.3±0.3		no
	ANSCO 1.0 g/kg UV-r	6.7±0.2		no
	ANSCO 1.0 g/kg UV-t	4.6±0.5		yes
	CBBO 0.3 g/kg UV-r	21±3		no
	CBBO 0.3 g/kg UV-t	6.7±2.4		yes
	CBBO 1.0 g/kg UV-r	59±4		no
	CBBO 1.0 g/kg UV-t	32±11		yes
Mar 18 Experiment			11–138	
	CBBO 0.3 g/kg UV-r	14±2		no
	CBBO 0.3 g/kg UV-t	9.3±0.7		yes
	CBBO 1.0 g/kg UV-r	29±2		no
	CBBO 1.0 g/kg UV-t	23±4		yes
Barron et al., 2003^2^			330–4200	
	ANSCO egg exposure	27		no
	ANSCO larval exposure	236		yes

1 Measured values and estimated LC_50_s are ng/g wet weight.

2 Reference [10].

Photomodification of PACs in solution is a consumptive process, with reduction of parent compound concentration as it is converted to oxidized derivatives. For example, a molar equivalent of anthracene was almost completely converted to quinones and other oxidized derivatives over the course of 10 hours of UV exposure [21]. To determine if photomodification was highly active in our oiled gravel effluents, we measured dissolved PACs at both the start and end of the 8-day incubation period. While there were somewhat lower concentrations of dissolved PACs in effluent samples at the end of incubation relative to the start of incubation (Fig. S5), there was no difference between effluents from UV-reducing and UV-transmitting treatments. Reduction of dissolved PAC concentrations over time under both conditions is consistent with the normal weathering (i.e. water-washing) process [15], while an absence of UV-dependent loss of parent compounds indicates that photomodification was not highly active.

The influence of elevated temperature as an additional stressor was tested inadvertently. Diurnal temperature spikes occurred during the course of the experiment, but they were not sufficient to explain the increased late embryonic mortality in CBBO-exposed embryos. The mid-day flow of treatment water through the columns produced temperature fluctuations in the incubation reservoirs, despite their submergence in the larger 12°C-cooling bath (Fig. S6). In the 26 February experiment the average temperatures of each logged reservoir ranged from 12.4 to 12.7°C with peak temperatures as high as 24.5°C. Increased column flow rates in the 18 March experiment resulted in lower average temperatures (10.4 to 10.6°C) with peak temperatures reaching 21.1°C. Two of the six incubation tables had overall lower peak temperatures during the 26 February experiment, and yet there was no correlation with the incidence of necrotic late embryos (Table S2). For example, CBBO-induced mortality was 90% in the water with the consistently lowest peak temperatures (water table 6), and 86% in the table with the consistently highest peak temperature (water table 4). Similarly, mortality rates in clean and unoiled urban gravel exposures were similar to those in laboratory incubator controls, despite temperatures ≥18°C on 6 days. Finally, we characterized the morphological response of hatching stage herring embryos to lethal temperature elevation (Fig. S7). Incubation on a heated microscope stage did not result in morphological changes or lethality until temperature well approached 35°C. The earliest response was in somitic muscle, which took on a coagulated appearance (Fig. S7A, B), causing distortion of the body axis (Fig. S7C). However, the central nervous system remained translucent, and the epidermis remained intact (Fig. S7D).

### Conclusions

At low doses of UV radiation, Cosco Busan bunker oil produced canonical and sublethal petrogenic PAC cardiotoxicity in herring embryos, represented by cardiogenic edema. These effects were significant at aqueous ∑PAC concentrations of 0.5 ppb, from very lightly oiled gravel (0.3 g/kg), which is unlikely to be characterized as visibly oiled in a post-spill shoreline survey. Thus, in the absence of sunlight, CBBO toxicity resembles previous observations of crude oil toxicity that is largely attributable to the tricyclic PACs, such as the phenanthrenes. Consistent with this, ANSCO exposure produced a lower incidence of pericardial edema than a mass-equivalent CBBO exposure that contained a 2.3-fold higher tricyclic PAC content. These findings are consistent with the observations of increased incidence of reduced heart rate and pericardial edema in embryos that were protected from sunlight in the subtidal zone at Cosco Busan oiled sites in 2008 [1].

In sharp contrast, CBBO in the presence of natural sunlight produced a novel form of lethal toxicity that would not be predicted based on the known toxicity of an equivalent mass loading of unrefined crude oil. Irrespective of the precise mechanism, our findings unequivocally demonstrate that combined exposure to weathered CBBO and sunlight, as would be expected to occur post-spill in shallow intertidal zones, is both necessary and sufficient to produce an acutely lethal necrosis in late-stage herring embryos that closely mimics the condition of embryos collected at oiled sites in San Francisco Bay in 2008 [1]. Importantly, these effects are virtually indistinguishable from our previous microscopic observations of tissue disintegration in zebrafish embryos caused by a photosensitization reaction from exposure to unweathered bunker oil followed by sunlight [11]. Due to the logistical difficulties of obtaining the large quantities of herring gametes necessary to produce a sufficient mass of embryos for both biological and chemical analysis (e.g., 2 g of embryos for each PAC determination), we chose a simple experimental design in which embryos were simultaneously exposed to oil and sunlight, in contrast to a sequential exposure design followed in our zebrafish studies. Given the scale and replication required for this study, it was not feasible to test all possible combinations of oil and sunlight exposure. Consequently, our design potentially could not formally distinguish between toxicity due to photomodification (e.g. sunlight-induced oxidation of compounds in oiled gravel effluent, followed by embryo uptake) or to photosensitization (i.e. uptake of parent compounds dissolved in oiled gravel effluent by embryos, followed by UV excitation). However, there was no chemical evidence of photomodification [21], as there was no UV-dependent loss of parent PACs from oiled gravel effluents during the incubation period.

Although embryos were also exposed to elevated temperatures for brief periods, this is insufficient to explain the necrotic response or elevated mortality. The average temperatures (10–12°C) were well within the range for normal development of San Francisco Bay herring, which have a slightly higher temperature tolerance than more northern populations (e.g. Puget Sound, Washington, USA) [22]. Incubation of herring embryos continuously at high temperatures (e.g. 15–20°C) resulted in elevated embryonic mortality and reduced hatching [22], but our observations on the morphological effects of acute lethal temperature stress demonstrated that loss of tissue integrity does not accompany elevated temperature. Moreover, control embryos exposed to clean or urban gravel effluent were exposed to the same temperature extremes, but did not suffer elevated mortality.

Overall, our findings in both herring embryos with weathered oil and zebrafish embryos with unweathered oil are consistent with the presence of photosensitizing compounds in bunker oils that produces a cellular phototoxic response that is unlike any previously described. The morphological effects observed in both cases involved a rapid loss of cellular integrity and disintegration of tissues. To our knowledge, there are only two other studies that documented the morphological changes in fish embryos that follow photosensitization reactions from PACs or other compounds. Embryos from fathead minnows (*Pimephales promelas*) that were maternally exposed to the phototoxic PAC anthracene followed by sunlight showed teratogenic effects (internal hemorrhaging, edema, and eye and yolk deformities), but not a necrotic response [23]. Medaka embryos exposed to higher concentrations of fluoranthene (5.6 µg/L, 28 nM) and intermittently exposed to UV light starting shortly after fertilization primarily showed failure of early development, with infrequent formation of a body axis [20]. Both of these responses are very different from our observation of a relatively normal progression through development followed by acute necrosis at the hatching stage. PAC photomodification products are unlikely to produce the rapid necrotic response observed here, findings that also support a photosensitization mechanism. PAC quinones have been shown to be weakly cytotoxic at micromolar concentrations [24], and are more likely to cause cell death by triggering apoptosis [25], [26], which would not lead to disintegration of tissues.

While the known phototoxic PACs (fluoranthene, pyrenes, and chrysenes) probably contribute to the lethal process observed here, the degree of phototoxicity cannot be explained by the tissue concentrations of these PACs alone. Indeed, none of the measured PACs can explain the phototoxicity of bunker oil, or even unrefined crude oil. A wide range of conventionally measured PACs has been tested for photoinduced toxicity in aquatic invertebrates, and quantitative structure-activity relationships (QSARs) developed for the biophysical properties associated with photoinduced toxicity [27], [28], but these studies are generally uninformative for the oils studied here. Both crude oil and its residual products contain mostly 2- and 3-ringed PACs, but lack the only tricyclic compounds with substantial phototoxicity, anthracene and its nitrogen heterocycle acridine [18], [27], [28], [29]. The remaining PACs with measured or predicted phototoxic potential are compounds with 4 or more rings that are either not detected in petroleum, or are not sufficiently water soluble to be bioavailable and detected in embryos exposed during oil weathering. In our previous analysis of bunker oil phototoxicity in zebrafish embryos, we identified 575 compounds in oil exposure water that included alkanes, monoaromatics, and PACs [11]. The most pronounced difference between the bunker oils (including CBBO) and unrefined ANSCO was the levels of 2- and 3-ring heterocyclic PACs, such as carbazoles, which are not predicted to have phototoxic activity in QSAR studies, and confirmed in tests here. None of the other 575 compounds were phototoxic candidates, other than the aforementioned PACs (fluoranthenes, pyrenes and chrysenes).

Although the phototoxicity of both CBBO and higher concentrations of unrefined crude oil (e.g. [10]) cannot be explained by known and conventionally measured PACs, the active compounds are most likely present in crude oil, and are simply more concentrated in residual fuel oils. Our results highlight how poorly characterized crude oil and products like residual fuel oil remain, both with respect to the myriad chemical constituents and their biological activities. Identification of the compounds underlying the effects described here will require a major effort using novel approaches in analytical chemistry. Methods, such as two-dimensional gas chromatography and two-dimensional gas chromatography/time-of-flight mass spectrometry, have shown promise in terms of further identifying compounds in petroleum products [30], [31], particularly in the “unresolved complex mixture”, which is typically a larger component of residual fuels oils (including CBBO). Similarly, detailed analysis of crude oil photomodification products has revealed the presence of previously uncharacterized parent PACs [32]. However, these chemical approaches have yet to be fully integrated into a program assessing biological effects.

The interaction between sunlight and an as-yet unidentified component of residual fuel oils has important implications for future spill assessments in sunlit aquatic habitats worldwide, particularly for forage fish and other species with translucent life stages. Bunker oil spills are likely to become increasingly common with expanding global shipping, and the ability to link biological impacts to chemical exposure as required in natural resource injury assessment cases will only be improved through more detailed characterizations of these diverse petroleum products.

## Materials and Methods

### Preparation of oiled gravel columns

Oiled gravel was prepared by tumbling in a portable cement mixer using a modification of previously published methods [33]. Locally obtained landscaping gravel was washed on 1-cm plastic sieves, and spread into monolayers on cardboard sheet to dry. Final drying was achieved with a heat gun. Aliquots of each oil (∼20 mL) were briefly held in brown glass bottles at 65°C in a water bath to maintain fluidity. A 5-mL glass pipet was calibrated to deliver desired masses of oil by adding oil drop-wise to a tared 25-mL beaker on an analytical balance. The number of drops required to deliver 1 g of oil was calibrated in triplicate and (52 drops for ANSCO, 47 for CBBO). For the lowest doses (0.1 g/kg and 0.3 g/kg) oil was scattered drop-wise over gravel in the mixer drum. Gravel was oiled in 11-kg batches using a separate mixer for each oil, from the lowest loading to the highest. For ANSCO at 0.1 g/kg, 54 drops (1.1 g) were added before tumbling. For CBBO at 0.1 g/kg, drops were added in three groups (16, 16, and 17; 1.1 g total) with brief tumbling between. For the CBBO 0.3 g/kg dose, 147 drops were added in five groups with tumbling between each. For the 1.0 g/kg doses, heated oil was weighed into a tared beaker, and poured onto tumbling gravel. The beaker was re-weighed after pouring to ensure delivery of 11 g total. Each batch of gravel was tumbled for 10 minutes after all oil was added. Gravel was spread out on aluminum foil-covered cardboard to dry up to 12 hours before packing into columns. Each dose of gravel was divided equally among 6 replicate columns (1-L glass beakers, 1.8 kg gravel each). Columns were covered with aluminum foil and stored indoors at room temperature until used.

Two of three experiments included two negative controls; clean gravel of the same batch used to generate oiled gravel, and gravel collected from a San Francisco Bay beach outside of the spill zone (“urban” gravel). Gravel of similar grain size was selected from the beach at China Camp State Park on the north side of Point San Pablo. The urban gravel was processed in the same way as the commercially obtained gravel.

### Exposure of herring embryos to column effluents

Exposures were conducted outdoors on a south-facing concrete pad. Six water tables to hold eight columns each were constructed in a terraced array to prevent tables from shading each other. Ambient full-strength seawater was mixed with fresh water to 22 psu salinity (“treatment water”) in a 3800-L holding tank. Fine clay silt present in the laboratory's well-water supply was removed with a 5-micron bag filter. Treatment water was delivered from the holding tank via a manifold to a peristaltic pump (Masterflex L/S variable speed drive with 8-channel cartridge, Cole-Parmer, Vernon Hills, IL) beneath each table, which distributed water to each of eight columns at a rate of 0.8 to 1 L/hr. Water was pumped into the bottom of each column via a tube (a section of 5-mL borosilicate glass pipet), up-welled over the lip of the beaker and was collected in custom made 20×41×8 cm aquaria serving as embryo exposure reservoirs (Fig. S1). A standpipe in each aquarium held the steady-state volume at 4-L. Temperature in the exposure aquaria was controlled by flooding each water table with seawater held at or below 12°C.

Three replicates of each oil dose and control gravel were weathered either under a cover of UV-transmitting plastic or UV-blocking plastic (to allow exposure to visible sunlight wavelengths). Covers were designed to block typical southerly or northerly rainfall, but were open on either end to allow air circulation and prevent heat trapping. Columns were randomly distributed by dose and oil type across all the tables, and half of each table (e.g. four columns) was randomized for a UV-blocking or UV-transmitting cover. Water flow to the columns was initiated 14 January 2009.

Herring gametes were dissected from freshly captured (via cast net or mid-water trawl) ripe adults and fertilized as described elsewhere [34]. Test fertilizations were conducted with eggs from individual females, and those with high fertilization success (≥90%) were pooled for large-scale fertilizations. Mean (± SEM) female weights for each experiment were 103.6±4.6 g (n = 5), 61.2±2.8 g (n = 33), and 62.7±4.8 (n = 13). Milt was pooled from five males for each experiment. Eggs were kept from clumping prior to fertilization with polyvinyl alcohol [34], and were distributed onto two substrates for exposure. For morphological observations, eggs were deposited onto frosted microscope slides targeting ∼100 eggs per slide. For analytical chemistry samples, 2–3 grams of eggs were deposited onto 10×20 cm sheets of nylon mesh. Embryos were distributed to exposure aquaria within 2 hours of fertilization and incubated to 8 dpf. During the first experiment (22 January), embryos were subsampled daily starting at 5 dpf to assess morphological effects of exposure, and determine the final assay time point for the subsequent experiments.

### Water quality monitoring and environmental data

During incubation, daily water quality measurements collected manually included temperature, salinity and dissolved oxygen. In addition, the aquarium for one control column (e.g. clean or urban gravel) on each table contained a continuous temperature probe that recorded every 10 minutes. Ammonia levels were measured colorimetrically for each aquarium before adding embryos and at the end of incubation. UV (290–390 nm) readings (Table 2) were collected daily under UV-transmitting plastic at the peak of solar radiation (between 1 and 2 PM) using a hand-held radiometer (Mannix UV-340, General Tools & Instruments, New York, NY). Paired measurements under UV-reducing and UV-transmitting plastics (Table S1) showed that UV was reduced by 58±5% (n = 8).

As day length and ambient temperature increased, the columns were colonized by algae, predominantly mat-forming benthic pennate diatoms (*Navicula* spp, *Nitzchia* spp, *Cylindrotheca* spp), with a few small flagellates and small filamentous macroalgae species. Algal growth during the 26 February and 18 March experiments produced alterations in dissolved oxygen and pH due to photosynthetic activity. Water in the incubation reservoirs became super-saturated with oxygen in a pattern that tracked with cloud cover, (e.g. Fig. S8A) and pH generally increased throughout the duration of incubation (Fig. S8B, D). Monitoring of dissolved oxygen through one day-night cycle indicated a rise and fall of super-saturation coincident with peak algal photosynthetic activity (data not shown). To reduce the levels of algae, 1 week prior to the 18 March experiment column flows were discontinued and the gravel emptied from each column into a sieve, gently washed of algae with fresh water, and re-packed. Lines and incubation reservoirs were treated with 10 ppm bleach, followed by deactivation with sodium thiosulfate. After extensive rinsing and testing for complete removal of bleach, seawater flow was restored 24 hours before the initiation of embryo incubation. Increased column flow rate resulted in better temperature control and reduced influence of algae on water chemistry. A comparison of water chemistry parameters between treatment groups by two-way ANOVA showed no significant differences, indicating that potential effects of elevated dissolved oxygen and pH were the same for all treatment groups.

### Zebrafish embryo exposures

Embryos of zebrafish AB strain were obtained from adults maintained using conventional zebrafish animal care protocols. NOAA's National Marine Fisheries Service does not require institutional review of animal care for fish studies, but we developed and published protocols for humane work with zebrafish based on those approved by the University of Washington's Institutional Animal Care and Use Committee [35]. Embryos were exposed to individual PACs and sunlight as described in detail elsewhere [11]. Briefly, stock solutions of individual PACs dibenzothiophene, carbazole, pyrene, fluoranthene, and chrysene (Sigma-Aldrich, St. Louis, MO) were made in DMSO and diluted into zebrafish culture water to 1 mg/L (∼50 µM for each compound). Embryos were exposed in 60-mm glass Petri dishes (25 embryos in 20 mL) in dark temperature-controlled incubators from a few hours to 24 hours post-fertilization. Embryos were then transferred into clean water in new Petri dishes, exposed to sunlight outdoors for 20 min, and returned to the laboratory and imaged as described elsewhere [11]. Control (no sunlight) embryos were exposed to the same outdoor ambient temperature under aluminum foil. Exposures were conducted in Seattle, Washington during the month of July. UV doses were not measured but would be comparable to previously published values [11].

### Analytical chemistry

Water samples were collected for PAC analysis on day 0 as embryos were added to aquaria, and at the end of exposures (day 8) using pre-cleaned 50-mL glass pipets and an automated filler. Only data from day 0 analyses are presented, and indicated the highest PAC concentration to which embryos were exposed. An additional water sample was obtained 13 February (Fig. S2), before an exposure that was aborted due to high rates of early gastrulation defects in the embryos in both columns and laboratory incubator controls. Before collecting samples from below the surface, an airstream was ejected from the pipet to displace any surface microlayer. 200-mL samples were collected from each aquarium with a fresh pipet, and stored in pre-cleaned 250-mL brown glass bottles with 20-mL methylene chloride. Day 0 samples were stored at 4°C until the end of the exposure when post-incubation samples were collected, then both sets were shipped to the NOAA Northwest Fisheries Science Center for analysis. Upon arrival samples were extracted with methylene chloride and analyzed using GC/MS selected ion monitoring as described previously with additional monitoring for alkylated PACs as described elsewhere [11], [36]. At the end of the exposures (day 8), embryos adhered to Nitex mesh sheets were collected from aquaria after displacing the surface with a glass “knife” (a 3-cm×10-cm glass sheet cut for each aquarium) to prevent potential contamination of eggs with surface oil sheen. Embryos were scraped with a stainless steel spatula into pre-cleaned jars (IChem) and frozen at −20°C. Embryo samples were shipped to Alpha Analytical, Inc. Woods Hole Division (Mansfield, MA) for extraction and PACs analysis.

### Microscopic examination of embryos and larvae and quantification of toxicity

Embryos and larvae were observed with oblique coherent contrast illumination using Nikon SMZ800 stereomicroscopes fitted with diascopic bases, and digital images captured using Fire-i 400 industrial cameras (Unibrain, Inc., San Ramon, CA) and BTV Pro 5.4.1 (www.bensoftware.com) on Apple PowerBook G4 computers. Embryos were held at 12°C using a temperature-controlled microscope stage (Brook Industries, Lake Villa, IL). Pre-hatch embryos were imaged without anesthesia either through the chorion or after manual dechorionation with fine forceps, while larvae were anesthetized with MS-222. In the first (22 January) experiment, embryos were subsampled, dechorionated, and examined in detail daily beginning at 5 dpf. During this experiment, cytolytic phototoxicity was observed by 8 dpf. Because this phenotype was readily visible through the chorion, and affected embryos did not remain intact with dechorionation, in subsequent experiments phototoxicity was quantified by counting cytolysed embryos through the chorions. In the 26 February and 18 March experiments, chorions were gently cleared of diatom coatings using forceps for unobstructed scoring of embryos. In the 26 February experiment, counts on one slide from each column included total eggs attached, unfertilized eggs, embryos that died during or before gastrulation, necrotic (cytolyzed) eyed embryos, and viable eyed embryos. Percentage of embryos showing necrotic phototoxicity was then normalized to total eyed embryos by subtracting unfertilized eggs and early lethal embryos from total eggs. In the 18 March experiment, counts for each slide included total eggs, unfertilized eggs, viable eyed embryos, and dead embryos irrespective of stage. Embryos that died during early development were quantified in the laboratory controls and averaged 13%. This value was indistinguishable from the rate of early mortality in the earlier experiments, where it was found that early mortality rates were independent of any treatment. The value for necrotic late embryos was obtained by subtracting the average early mortality (13%) from total mortality normalized to fertilized embryos. For scoring of edema, one replicate slide from each column was placed into 250 mL glass culture dishes containing 200 mL 16 ppt seawater and incubated in a 12°C incubator. Hatched larvae were collected daily up to 8 days after retrieval from column effluent (i.e. 15 dpf), anesthetized with MS-222 and examined microscopically for pericardial and yolk sac edema. Calculation of percent with edema was based on total hatched live larvae.

### Morphological assessment of acute temperature elevation

Dechorionated hatching-stage embryos were anesthetized with MS-222 and placed in filtered seawater in a 100-mm Petri dish and allowed to equilibrate to room temperature for 1 hour. After no effects were observed, the Petri dish was placed on the temperature-controlled stereomicroscope stage set to heating mode at 35°C. Images were collected immediately after the first morphological changes were observed.

## Supporting Information

Figure S1 **System for combined exposure to oiled gravel effluent and sunlight.** (A) Schematic overview showing design of randomized array of dosing columns. (B) Schematic detail of a single column/incubation reservoir unit. (C) Photo of actual experimental setup.
(TIF)

Figure S2 **Aqueous PAC concentrations in column effluents at three time points.** Data are from water samples taken 13 February, 26 February, and 18 March. For each treatment, bars represent the mean value ± s.e.m (three replicates) for each experiment, respectively. ANSCO and urban gravel columns were excluded from the 18 March experiment, so these treatments show only two bars. Blue bars are PAC concentrations from columns under UV-reducing plastic (*UV-r*), red bars are data for UV-transmitting plastic (*UV-t*). (A) Summed PAC concentrations. (B) Sum parent and alkyl-naphthalenes (NPHs). (C) Sum parent and alkyl-fluorenes (FLUs). ((D) Sum parent and alkyl-dibenzothiphenes (DBTs). (E) Sum parent and alkyl-phenanthrenes (PHNs). (F) sum high molecular weight (HMW) 4-, 5-, and 6-ring compounds.
(TIF)

Figure S3 **Composition of PACs in column effluents and exposed herring embryos for negative controls, middle and low doses of oiled gravel.** Composition of PACs determined by GC/MS represented as percent of summed PACs for each of 39 analytes (mean ± s.e.m. for three replicate columns). Data are shown only for UV reduced conditions from the 26 February experiment, but are representative of the general patterns observed for UV transmitting treatments and the 18 March experiment. Each plot shows PAC composition for effluent (blue bars) paired with embryo tissue (red bars). (A) Clean gravel control. (B) Urban gravel control. (C) ANSCO 0.1 g/kg dose. (D) ANSCO 0.3 g/kg dose. (E) CBBO 0.1 g/kg dose. (F) CBBO 0.3 g/kg dose. N, naphthalenes; AY, acenaphthylene; AE, acenaphthene; F, fluorene; D, dibenzothiophene; P, phenanthrene; A, anthracene; FL, fluoranthene; PY, pyrene; FP, fluoranthenes/pyrenes; BA, benz[*a*]anthracene; C, chrysene; BBF, benzo[*b*]fluoranthene; BJKF, benzo[*j*]fluoranthene/benzo[*k*]fluoranthene; BEP, benzo[e]pyrene; BAP, benzo[a]pyrene; PER, perylene; IND, indeno[1,2,3-*cd*]pyrene, DBA, dibenz[*a*,*h*]anthracene/dibenz[*a*,*c*]anthracene; BZP, benzo[*ghi*]perylene. Parent compound is indicated by a 0 (e.g., N0), while numbers of additional carbons (e.g. methyl groups) for alkylated homologs are indicated as N1, N2, etc.
(TIF)

Figure S4 **Herring embryos exposed to both oil types were viable at 6 dpf. Embryos at 6 dpf (late segmentation stage, about 50 somites) were manually dechorionated and imaged live as described in the** **Materials and methods** **.** Embryos were exposed to (A) ANSCO 1.0 g/kg under UV-reducing (UV-r) plastic; (B) ANSCO 1.0 g/kg under UV-transmitting (UV-t) plastic; (C) CBBO 1.0 g/kg under UV-reducing (UV-r) plastic; (D) CBBO 1.0 g/kg under UV-transmitting (UV-t) plastic; (E) Urban control gravel under UV-transmitting (UV-t) plastic. Double arrows (A–C, E) indicate the extent of the tail bud, from the urogenital pore to the posterior end; arrowheads (D) indicate UV-dependent tail bud deterioration. Scale bar is 1 mm.
(TIF)

Figure S5 **Lack of UV-dependent loss of parent PAC compounds from oiled gravel column effluents.** Dissolved concentrations (ng/L) of selected non-phototoxic and phototoxic PACs are shown for the high CBBO dose (1 g/kg) at the beginning and end of embryo incubations under UV-reducing (UV-r) and UV-transmitting (UV-t) plastic. Values are means and error bars are s.e.m. for three replicate columns. P, phenanthrene; FL, fluoranthene; PY, pyrene; FP, fluoranthenes/pyrenes; C, chrysene. Parent compound is indicated by a 0 (e.g., N0), while numbers of additional carbons (e.g. methyl groups) for alkylated homologs are indicated as P1, P2, etc.
(TIF)

Figure S6 **Diurnal temperature elevation in column effluents. Data are plots of continuous temperature recordings every 10 minutes for the duration of embryo incubation. (A) 26 February experiment.** Temperature was recorded in one negative control column (either clean or urban gravel) on each table. (B) 18 March experiment. Only three of six tables were used, and temperature was recorded in UV-reducing and UV-transmitting negative controls on each table.
(TIF)

Figure S7 **Morphological effects of acute temperature elevation in herring embryos.** Embryos were exposed to a microscope stage either heated to 35°C (A, C, D), or cooled to 10°C (B). Arrows in (A) and (B) indicate somites, which are normally chevron-shaped and translucent, revealing the notochord beneath (B; *nc*, double arrow). Coagulation and shrinkage of the muscle fibers following extreme temperature elevation altered the appearance of somites (A), and resulted in distortion of the body axis (examples in C). (D) Higher magnification of the head. Scale bars are 0.5 mm.
(TIF)

Figure S8 **Changes in dissolved oxygen (DO) and pH due to algal growth during embryo incubation.** Daily DO (as percent saturation) and pH measurements are plotted for duration of embryo incubation for the 26 February experiment (A, C) and 18 March experiment (B, D). Measurements for clean controls and CBBO 1.0 g/kg doses (26 February experiment) and two replicates of clean controls and each CBBO dose (18 March experiment) are color-coded for each DO/pH pair. (E) Average (± SEM) DO from 8 columns on water table 5 showed daytime supersaturation with a nocturnal trough.
(TIF)

Table S1 **Level of UV reduction by UV blocking plastic was assessed using a hand-held radiometer as described in the** **Materials and methods** **section.**
(DOC)

Table S2 **Average and peak temperature measures for each water table were obtained from continuous temperature loggers (plots shown in Figure S6A) and compared to individual mortality levels for replicates of the given treatment group incubated in each table (contributing to the average values shown in** **Figure 6A** **.**
(DOC)
