# Supplementary figures and images for: Potent Phototoxicity of Marine Bunker Oil to Translucent Herring Embryos after Prolonged Weathering
Source: PLoS One. 2012 Feb 1;7(2):e30116. doi: 10.1371/journal.pone.0030116 (PMC3270018; doi:10.1371/journal.pone.0030116)

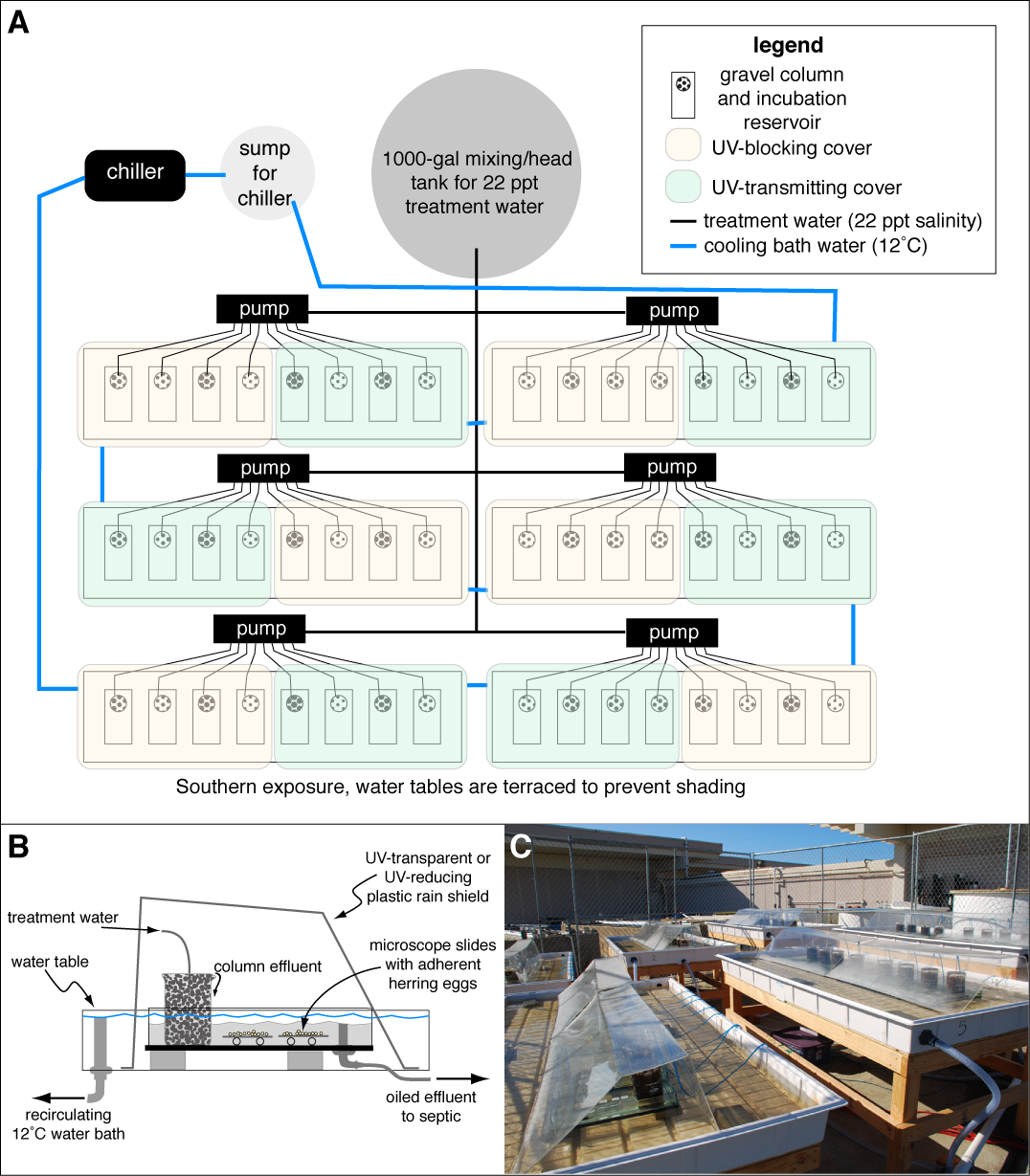

Supplement: Figure S1 — System for combined exposure to oiled gravel effluent and sunlight. (A) Schematic overview showing design of randomized array of dosing columns. (B) Schematic detail of a single column/incubation reservoir unit. (C) Photo of actual experimental setup. (TIF) [file pone.0030116.s001.tif]

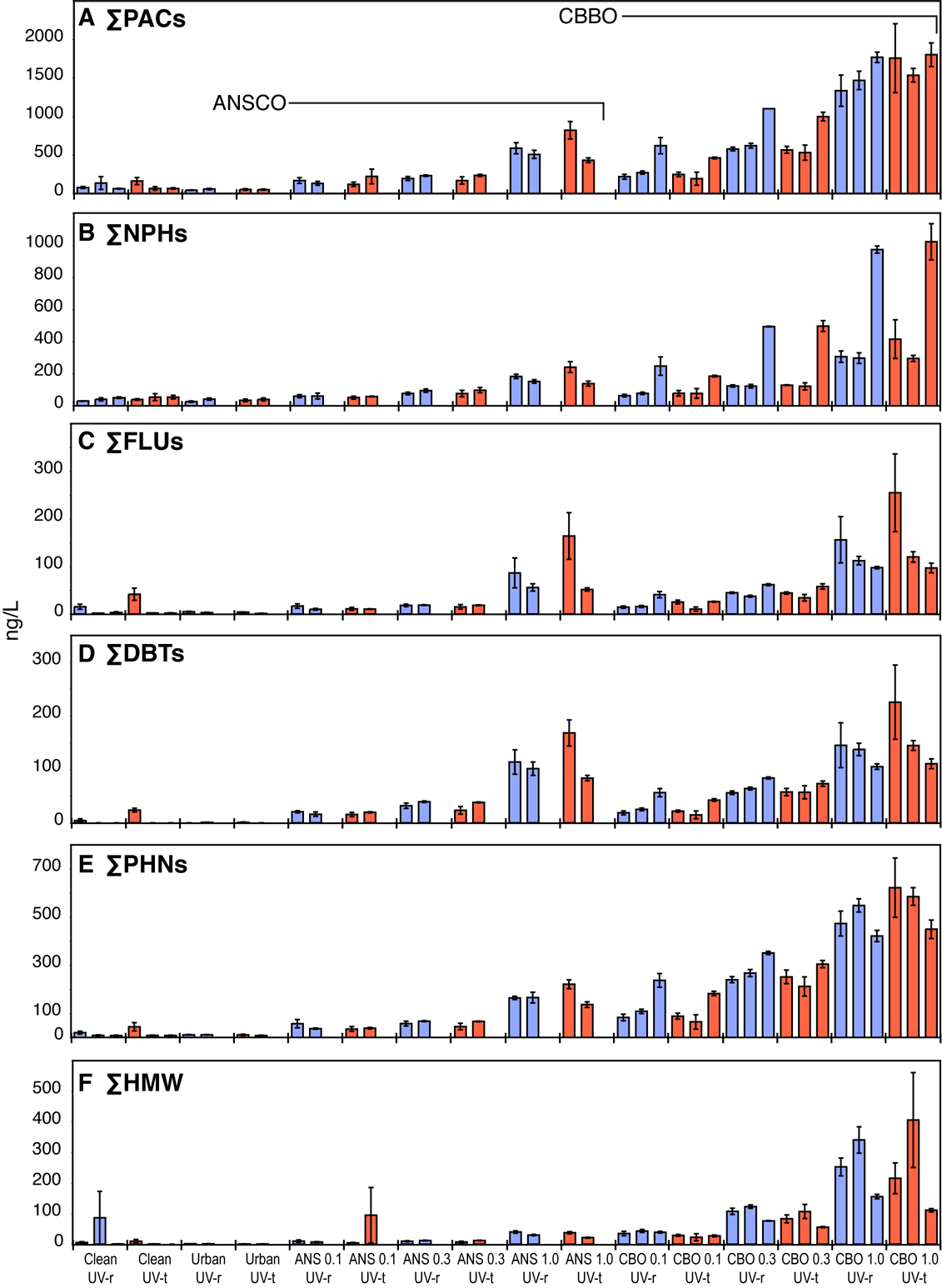

Supplement: Figure S2 — Aqueous PAC concentrations in column effluents at three time points. Data are from water samples taken 13 February, 26 February, and 18 March. For each treatment, bars represent the mean value ± s.e.m (three replicates) for each experiment, respectively. ANSCO and urban gravel columns were excluded from the 18 March experiment, so these treatments show only two bars. Blue bars are PAC concentrations from columns under UV-reducing plastic (UV-r), red bars are data for UV-transmitting plastic (UV-t). (A) Summed PAC concentrations. (B) Sum parent and alkyl-naphthalenes (NPHs). (C) Sum parent and alkyl-fluorenes (FLUs). ((D) Sum parent and alkyl-dibenzothiphenes (DBTs). (E) Sum parent and alkyl-phenanthrenes (PHNs). (F) sum high molecular weight (HMW) 4-, 5-, and 6-ring compounds. (TIF) [file pone.0030116.s002.tif]

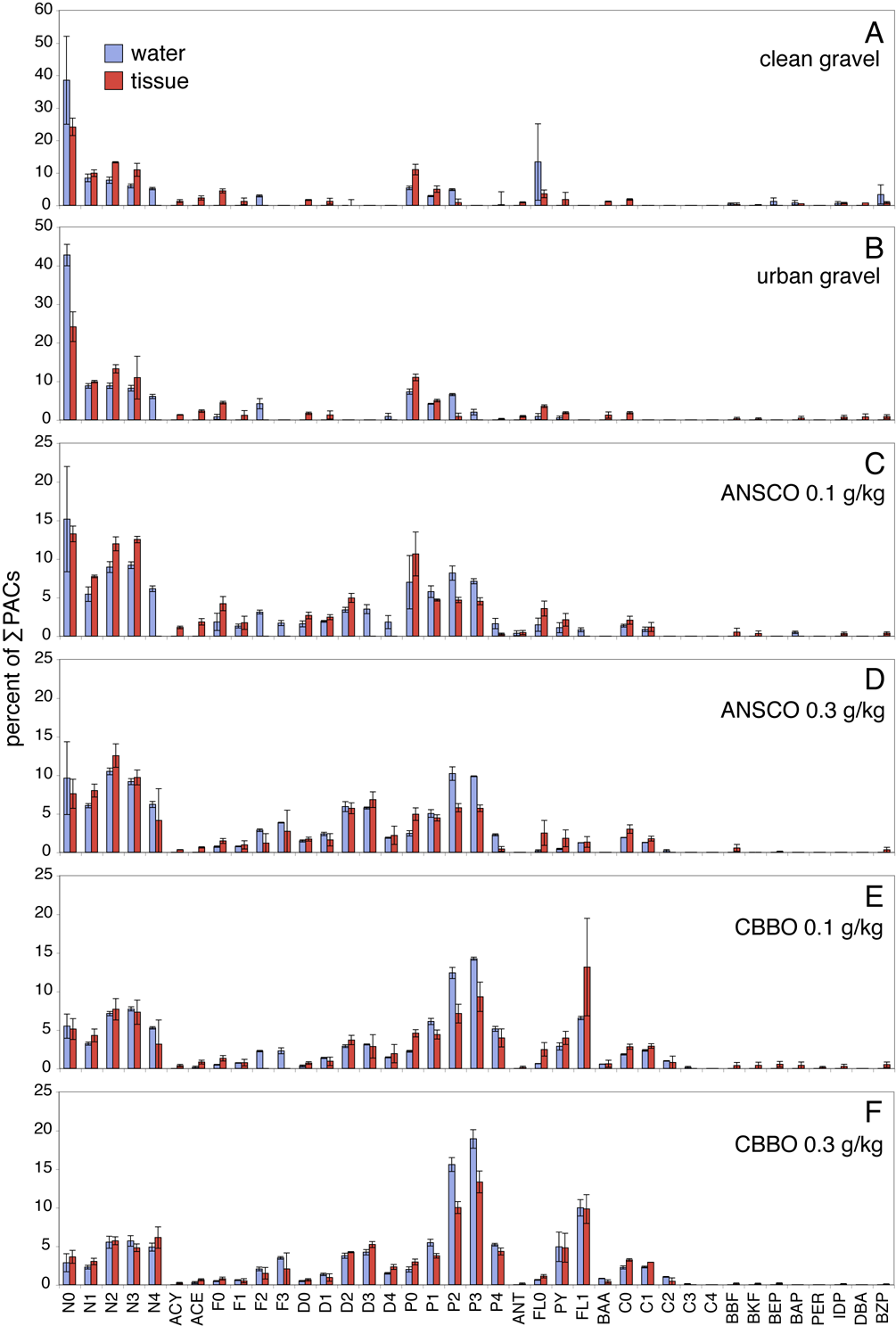

Supplement: Figure S3 — Composition of PACs in column effluents and exposed herring embryos for negative controls, middle and low doses of oiled gravel. Composition of PACs determined by GC/MS represented as percent of summed PACs for each of 39 analytes (mean ± s.e.m. for three replicate columns). Data are shown only for UV reduced conditions from the 26 February experiment, but are representative of the general patterns observed for UV transmitting treatments and the 18 March experiment. Each plot shows PAC composition for effluent (blue bars) paired with embryo tissue (red bars). (A) Clean gravel control. (B) Urban gravel control. (C) ANSCO 0.1 g/kg dose. (D) ANSCO 0.3 g/kg dose. (E) CBBO 0.1 g/kg dose. (F) CBBO 0.3 g/kg dose. N, naphthalenes; AY, acenaphthylene; AE, acenaphthene; F, fluorene; D, dibenzothiophene; P, phenanthrene; A, anthracene; FL, fluoranthene; PY, pyrene; FP, fluoranthenes/pyrenes; BA, benz[a]anthracene; C, chrysene; BBF, benzo[b]fluoranthene; BJKF, benzo[j]fluoranthene/benzo[k]fluoranthene; BEP, benzo[e]pyrene; BAP, benzo[a]pyrene; PER, perylene; IND, indeno[1,2,3-cd]pyrene, DBA, dibenz[a,h]anthracene/dibenz[a,c]anthracene; BZP, benzo[ghi]perylene. Parent compound is indicated by a 0 (e.g., N0), while numbers of additional carbons (e.g. methyl groups) for alkylated homologs are indicated as N1, N2, etc. (TIF) [file pone.0030116.s003.tif]

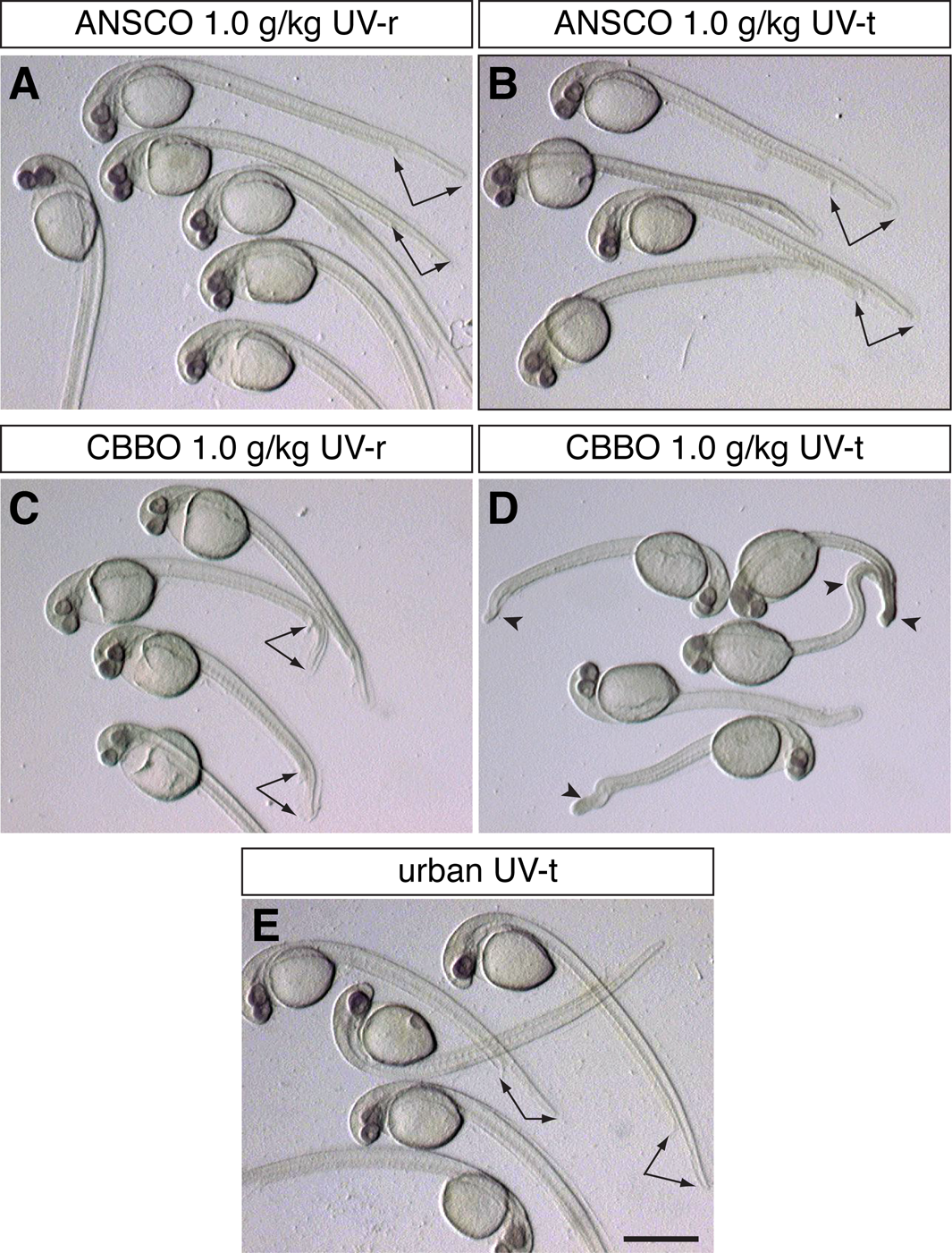

Supplement: Figure S4 — Herring embryos exposed to both oil types were viable at 6 dpf. Embryos at 6 dpf (late segmentation stage, about 50 somites) were manually dechorionated and imaged live as described in the Materials and methods . Embryos were exposed to (A) ANSCO 1.0 g/kg under UV-reducing (UV-r) plastic; (B) ANSCO 1.0 g/kg under UV-transmitting (UV-t) plastic; (C) CBBO 1.0 g/kg under UV-reducing (UV-r) plastic; (D) CBBO 1.0 g/kg under UV-transmitting (UV-t) plastic; (E) Urban control gravel under UV-transmitting (UV-t) plastic. Double arrows (A–C, E) indicate the extent of the tail bud, from the urogenital pore to the posterior end; arrowheads (D) indicate UV-dependent tail bud deterioration. Scale bar is 1 mm. (TIF) [file pone.0030116.s004.tif]

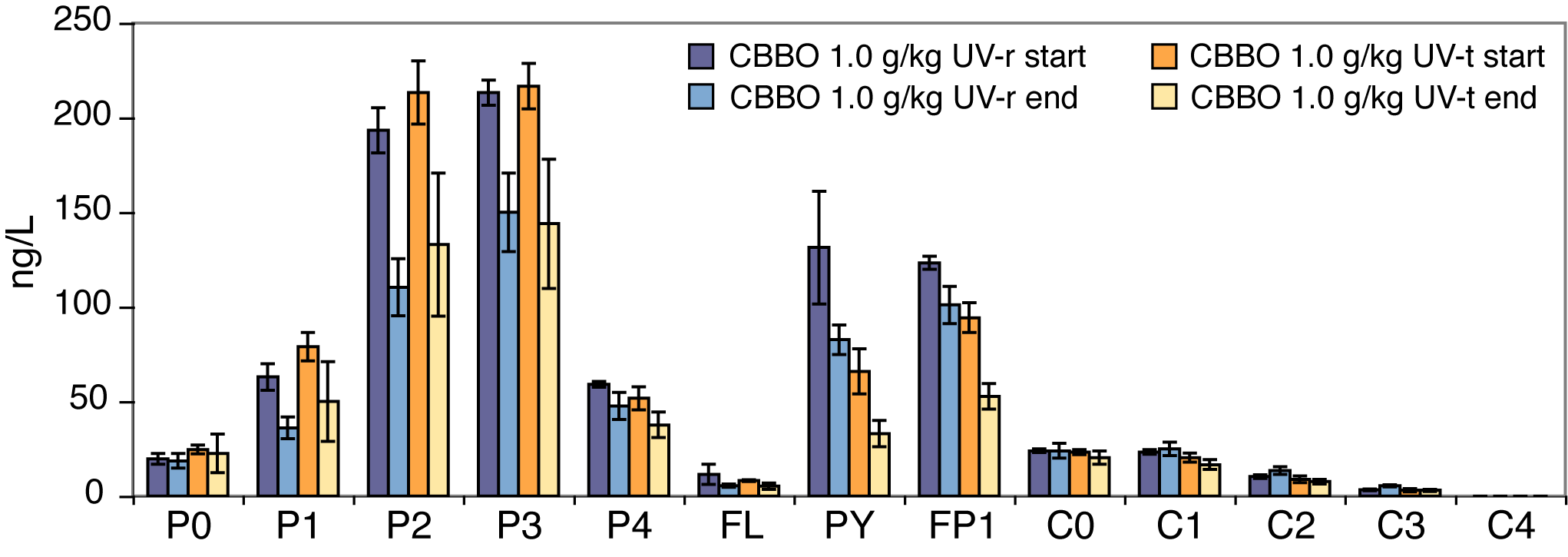

Supplement: Figure S5 — Lack of UV-dependent loss of parent PAC compounds from oiled gravel column effluents. Dissolved concentrations (ng/L) of selected non-phototoxic and phototoxic PACs are shown for the high CBBO dose (1 g/kg) at the beginning and end of embryo incubations under UV-reducing (UV-r) and UV-transmitting (UV-t) plastic. Values are means and error bars are s.e.m. for three replicate columns. P, phenanthrene; FL, fluoranthene; PY, pyrene; FP, fluoranthenes/pyrenes; C, chrysene. Parent compound is indicated by a 0 (e.g., N0), while numbers of additional carbons (e.g. methyl groups) for alkylated homologs are indicated as P1, P2, etc. (TIF) [file pone.0030116.s005.tif]

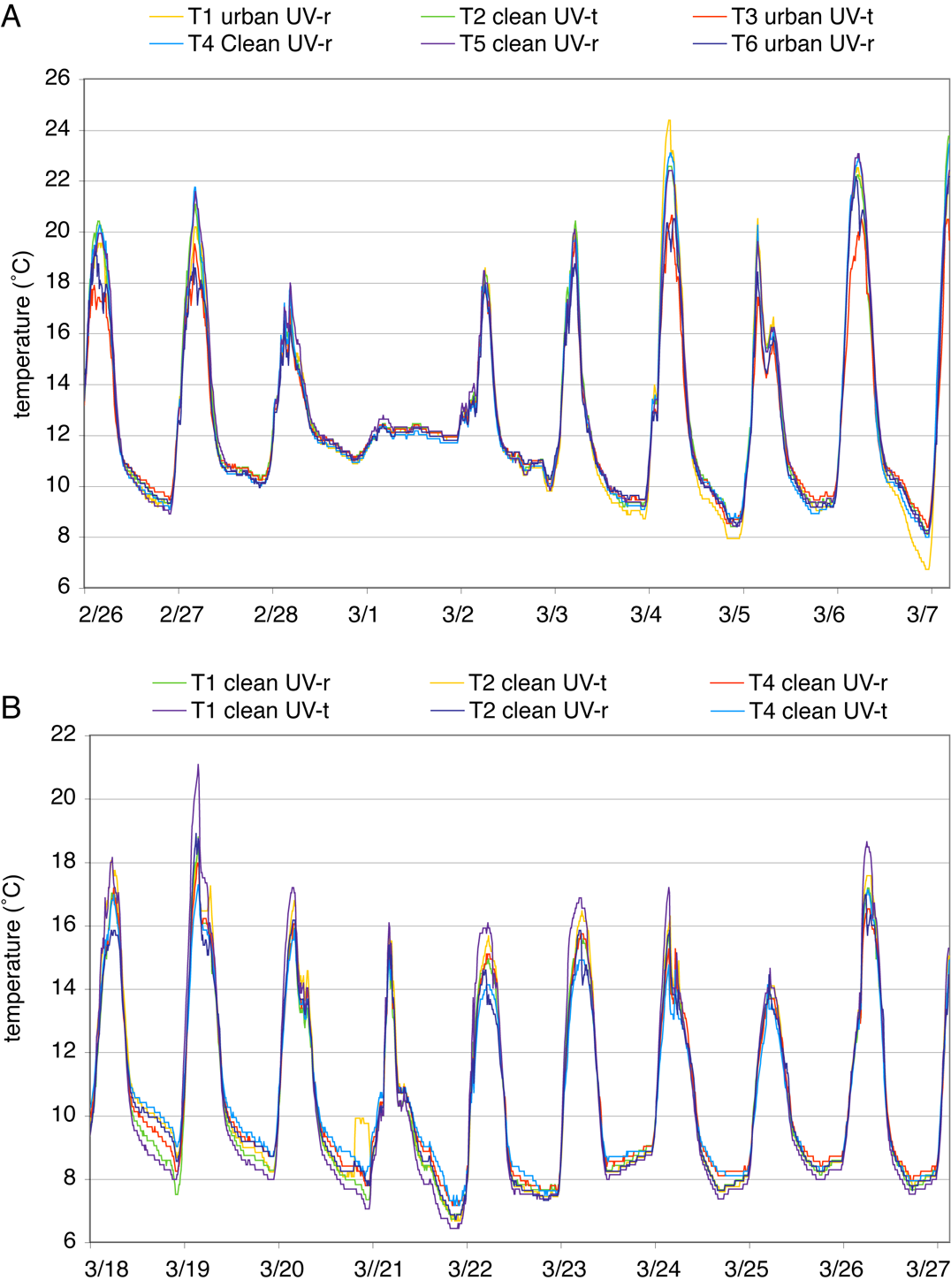

Supplement: Figure S6 — Diurnal temperature elevation in column effluents. Data are plots of continuous temperature recordings every 10 minutes for the duration of embryo incubation. (A) 26 February experiment. Temperature was recorded in one negative control column (either clean or urban gravel) on each table. (B) 18 March experiment. Only three of six tables were used, and temperature was recorded in UV-reducing and UV-transmitting negative controls on each table. (TIF) [file pone.0030116.s006.tif]

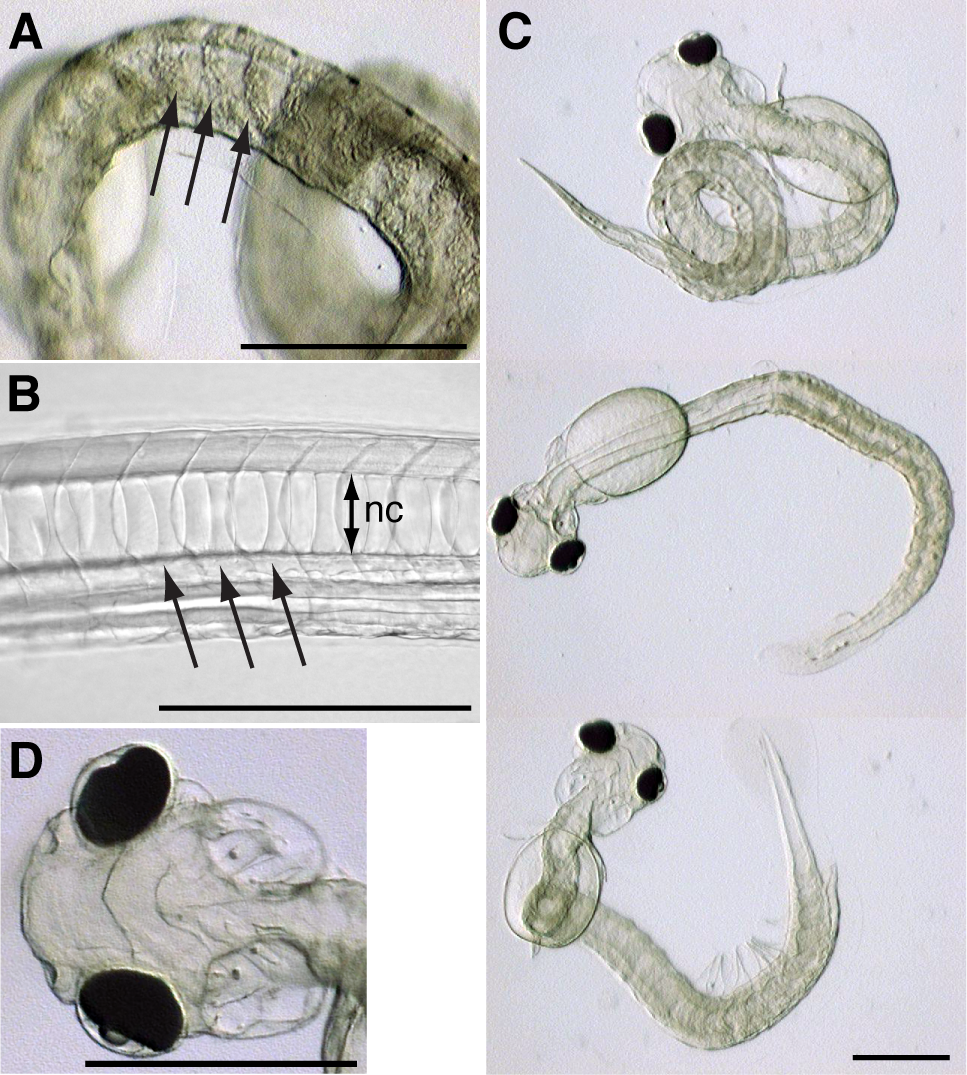

Supplement: Figure S7 — Morphological effects of acute temperature elevation in herring embryos. Embryos were exposed to a microscope stage either heated to 35°C (A, C, D), or cooled to 10°C (B). Arrows in (A) and (B) indicate somites, which are normally chevron-shaped and translucent, revealing the notochord beneath (B; nc, double arrow). Coagulation and shrinkage of the muscle fibers following extreme temperature elevation altered the appearance of somites (A), and resulted in distortion of the body axis (examples in C). (D) Higher magnification of the head. Scale bars are 0.5 mm. (TIF) [file pone.0030116.s007.tif]

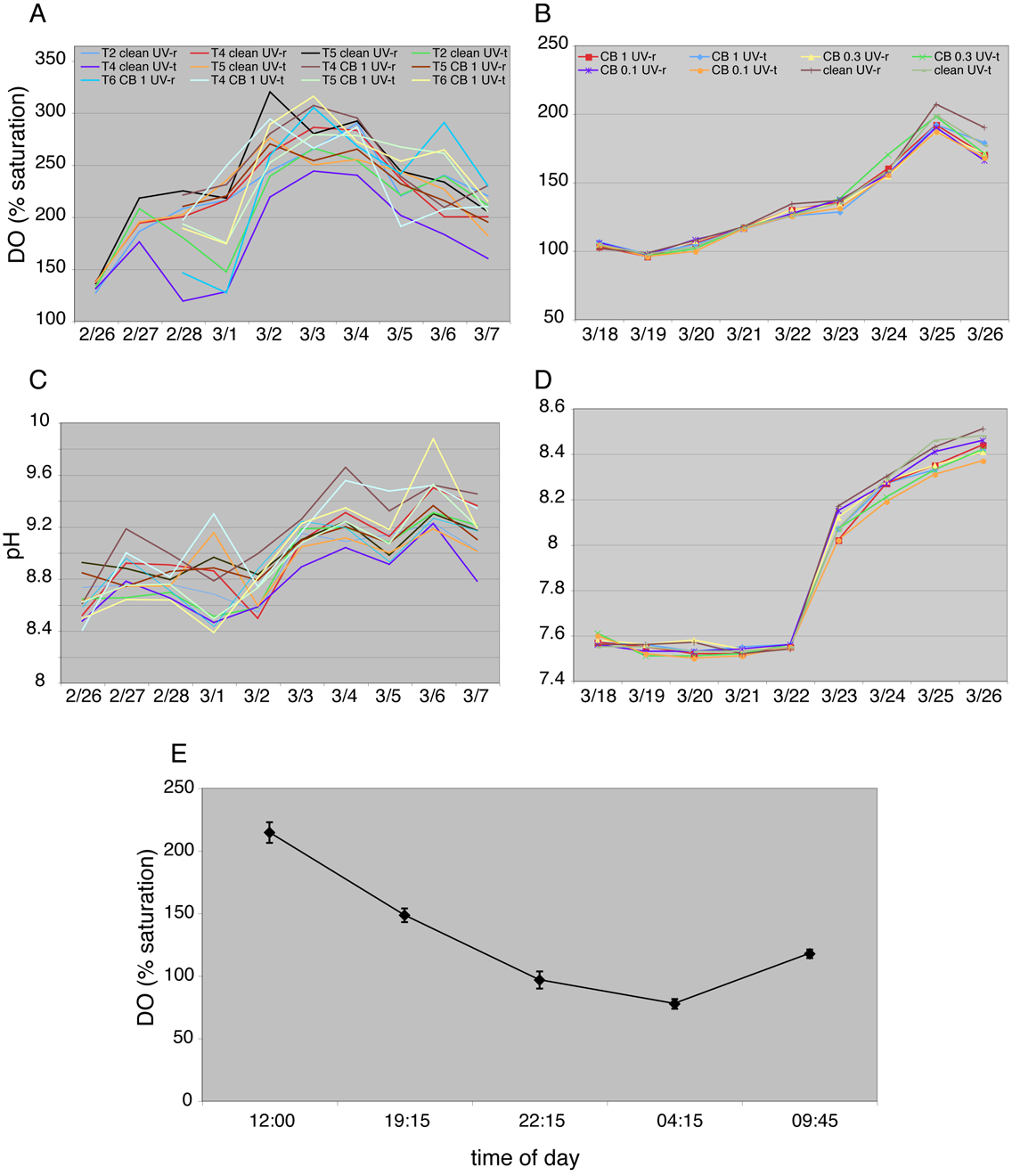

Supplement: Figure S8 — Changes in dissolved oxygen (DO) and pH due to algal growth during embryo incubation. Daily DO (as percent saturation) and pH measurements are plotted for duration of embryo incubation for the 26 February experiment (A, C) and 18 March experiment (B, D). Measurements for clean controls and CBBO 1.0 g/kg doses (26 February experiment) and two replicates of clean controls and each CBBO dose (18 March experiment) are color-coded for each DO/pH pair. (E) Average (± SEM) DO from 8 columns on water table 5 showed daytime supersaturation with a nocturnal trough. (TIF) [file pone.0030116.s008.tif]
