# Supplementary material for: Potent Phototoxicity of Marine Bunker Oil to Translucent Herring Embryos after Prolonged Weathering
Source: PLoS One. 2012 Feb 1;7(2):e30116. doi: 10.1371/journal.pone.0030116 (PMC3270018; doi:10.1371/journal.pone.0030116)
Supplement: Table S1 — Level of UV reduction by UV blocking plastic was assessed using a hand-held radiometer as described in the Materials and methods section. (DOC) [file pone.0030116.s009.doc]

**Table S1: Reduction of UV levels by UV blocking plastic.**

|  |  | **radiometer readings (µW/cm^2^)** | | | **percent block** | |
| --- | --- | --- | --- | --- | --- | --- |
| **Date** | **time** | **no plastic** | **UV transparent plastic** | **UV reducing plastic** | **UVB/UVT** | **UVT/no plastic** |
| 1/29/09 | 9:00 | 1600 | 1400 | 700 | 50.0 | 12.5 |
| 1/29/09 | 11:37 | 540 | 420 | 250 | 40.5 | 22.2 |
| 1/29/09 | 13:00 | 790 | 770 | 390 | 49.4 | 2.5 |
| 1/29/09 | 14:00 | 730 | 680 | 260 | 61.8 | 6.8 |
| 1/29/09 | 15:10 | 570 | 560 | 160 | 71.4 | 1.8 |
| 1/29/09 | 16:30 | 150 | 140 | 30 | 78.6 | 6.7 |
| 1/30/09 | 9:40 | 450 | 580 | 220 | 62.1 | -28.9 |
| 1/30/09 | 12:15 | 870 | 840 | 430 | 48.8 | 3.4 |
| mean ± sem |  |  |  |  | 57.8 ± 4.6 | 3.4 ± 5.2 |
