# Supplementary material for: Potent Phototoxicity of Marine Bunker Oil to Translucent Herring Embryos after Prolonged Weathering
Source: PLoS One. 2012 Feb 1;7(2):e30116. doi: 10.1371/journal.pone.0030116 (PMC3270018; doi:10.1371/journal.pone.0030116)
Supplement: Table S2 — Average and peak temperature measures for each water table were obtained from continuous temperature loggers (plots shown in Figure S6A) and compared to individual mortality levels for replicates of the given treatment group incubated in each table (contributing to the average values shown in Figure 6A . (DOC) [file pone.0030116.s010.doc]

**Table S2: Average and peak temperatures do not correlate with embryonic mortality**

| **table** | **mean temp** | **peak temperature^1^** | | | | | | | | | | **percent late embryonic lethality** | | | |
| --- | --- | --- | --- | --- | --- | --- | --- | --- | --- | --- | --- | --- | --- | --- | --- |
|  |  | 2/26 | 2/27 | 2/28 | 3/1 | 3/2 | 3/3 | 3/4 | 3/5 | 3/6 | 3/7 | CB 1 UV-r | CB 1 UV-t | clean UV-r | clean UV-t |
| T1 | 12.5 | 19.5 | 20.2 | 17 | ***12.4*** | 18.6 | 20 | **24.4** | 20.5 | 22.5 | 23 | - | - | - | - |
| T2 | 12.7 | **20.4** | 21.1 | 17.3 | ***12.4*** | 18.4 | 20.4 | 22.6 | 19.9 | 22.2 | 24.1 | - | - | 5.0 | 2.0 |
| T3 | 12.4 | ***17.9*** | 19.5 | 16.9 | ***12.4*** | 18.0 | 19.7 | 20.6 | ***17.4*** | ***20.5*** | ***21.0*** | - | - | - | - |
| T4 | 12.6 | 20.3 | **21.7** | 17.8 | **18.0** | **19.8** | **23.1** | 23.1 | **22.9** | **24.5** | **24.5** | 13.0 | 86.0 | 0.0 | 5.0 |
| T5 | 12.7 | 19.9 | 21.6 | **18.0** | 12.8 | 18.5 | 20.1 | 22.4 | 19.6 | 23.1 | 22.9 | 23.0 | 97.0 | 3.0 | 0.0 |
| T6 | 12.5 | 19.2 | ***18.7*** | ***16.5*** | ***12.4*** | ***17.9*** | ***18.7*** | ***20.5*** | 18.4 | 22.2 | 23.0 | 4.0 | 90.0 |  |  |

^1^Lowest and highest peak temperatures indicated in bold italics and bold, respectively
